# The time-varying bidirectional causal relationship between household education expenditure and resident credit behavior: Dynamic quantile evidence and heterogeneous mechanisms

**DOI:** 10.1371/journal.pone.0329213

**Published:** 2025-08-13

**Authors:** Chunyan Jiang, Yayun Wang, Wanqi Li, Runze Ding

**Affiliations:** 1 School of Finance and Economics, Shenzhen Institute of Information Technology, Shenzhen, Guangdong Province, China; 2 School of Economics, Shenzhen Polytechnic University, Shenzhen, Guangdong Province, China; 3 Cheung Kong School of Journalism and Communication, Shantou University, Shantou, Guangdong Province, China; 4 School of Culture and Communication, Swansea University, Singleton Park, Swansea, United Kingdom; University of Professional Studies, GHANA

## Abstract

This study aims to investigate the time-varying bidirectional causal relationship between household education expenditure and resident credit behavior, as well as the heterogeneous mechanisms under different economic conditions and household characteristics. By constructing a TVP-SV-VAR model and a QVAR-DY model, we analyze urban household data in China from January 2015 to December 2024, unveiling the dynamic relationship between education expenditure and credit behavior, along with their asymmetry and heterogeneity. The findings reveal a significant bidirectional causal relationship between household education expenditure and resident credit behavior, which exhibits heterogeneity across different quantile levels and is influenced by household income, education level, and credit interest rates. Additionally, this study employs static and dynamic window methods to analyze the short-term, medium-term, and long-term spillover effects. Based on these findings, we propose policy recommendations for optimizing household education investment and credit market management under low, medium, and high-risk levels.

## 1. Introduction

In recent years, household education expenditure has been on a continuous upward trend as a vital part of household economic expenditure. With the socio-economy developing and families increasingly emphasizing education, the proportion of education expenditure in the total household budget has risen yearly. According to data from the National Bureau of Statistics of China, by 2019, education expenditure among urban households in China had accounted for over 15% of total household expenditure, with this proportion being even more pronounced in some developed regions. This reflects families’ heightened attention to their children’s education and reveals the significant role of education consumption in household financial decision-making.

Concurrently, as financial markets deepen and innovate, the resident credit market has become an important source of funding for household education expenditure. Financial instruments like education loans and credit card installment payments have been widely adopted, enabling families to arrange educational expenses more flexibly. However, this has also brought potential financial risks, such as the rising household debt ratio due to over-reliance on credit, which directly impacts family financial health and long-term economic security.

Prior studies have extensively discussed the impact of credit on educational consumption and the effect of educational expenditure on credit behavior. However, few have delved into the potential bidirectional causality and heterogeneous mechanisms between the two. Existing research mainly focuses on the one-way impact of credit on education expenditure or the reaction of credit behavior to education expenditure. However, their relationship is more complex, with possible mutual influence and different mechanisms under various conditions. For instance, the interaction between education expenditure and credit behavior may vary significantly in family income levels, education stages, or regional financial development levels.

To address this gap and meet the research need of comprehensively understanding this complex relationship, the present study employs dynamic quantile regression methods and a heterogeneity analysis framework to investigate the time-varying bidirectional causal relationship between household education expenditure and resident credit behavior, employs the TVP-SV-VAR and QVAR-DY methodologies analysis to comprehensively capture the bidirectional interaction between household education expenditure and resident credit behavior. This research is motivated by the practical need to help families better manage their finances in the context of rising education expenditure and widespread credit use, as well as the academic need to enrich the theoretical knowledge in household economics and behavioral finance by exploring the bidirectional and heterogeneous relationship between education expenditure and credit behavior. This study’s findings will help clarify the complex dynamics in household financial decision-making and provide empirical support for policymakers to develop more scientifically sound educational finance policies to safeguard family financial health and social and economic stability.

This study yields four key findings: First, a significant and dynamic two-way causal relationship exists between family educational expenditure and household lending behavior. Households borrow more to manage higher educational costs, and easier credit access boosts educational investment, yet the strength and stability of this relationship fluctuate over time. Second, pronounced heterogeneity appears across different risk levels. At low, medium, and high-risk levels, variables like educational consumption and household debt-to-income ratio exhibit varying spillover effects. Specifically, educational expenditure and household debt-to-income ratio are primary net spillover senders at low-risk levels, while household debt-to-income ratio shows more significant spillover effects at high-risk levels. Third, spillover effects are not static. Regardless of whether analyzed statically, dynamically, or across different time scales, the spillover effects between variables are dynamic. These effects and their interaction patterns differ under varying risk levels and economic conditions. Fourth, economic uncertainty has multifaceted impacts. When economic uncertainty arises, households initially cut educational expenditures to ease potential financial pressure, though this effect fades over time. Moreover, economic uncertainty affects households’ ability and willingness to obtain credit, further influencing market stability.

## 2. Literature review

### 2.1. Multidimensional exploration of household education expenditure: Current status and challenges

As a significant component of family resource allocation, household education expenditure is influenced by various factors, including but not limited to family structure, parental occupational status, socioeconomic position, and regional cultural background. This section aims to systematically review existing studies to explore the essential characteristics of household education expenditure and its determining factors, and to analyze how these factors influence the allocation of educational resources within families.

#### 2.1.1. Household resource allocation and educational expenditure decision-making.

Household resource allocation is influenced by a complex interplay of factors. In terms of family structure [1], found that homemakers in South Korean families significantly elevate extracurricular educational expenditures, reflecting the profound impact of gender division of labor on resource distribution [2] further indicated that sibling structure leads to uneven allocation of non-monetary resources, with girls facing educational opportunity deprivation due to increased domestic responsibilities. Socioeconomic status constitutes a core constraint: [3] confirmed that income levels directly determine the intensity of higher education investment, while [4] revealed that children of fishermen lose developmental opportunities due to economic disadvantages. In Turkey, high-income groups widen class disparities through educational expenditure expansion [5]. Notably, cognitive biases significantly shape investment behavior. Parents’ upward bias regarding their children’s academics increases educational investment by raising expectations [6], yet cash subsidies must be designed with incentive mechanisms to be effectively translated into investments [7]. Cultural capital, as an intermediary mechanism, must not be overlooked [8] and [9] confirmed that cultural capital, such as reading preferences, transmits educational inequality [10] discovered that Islamic values guide families to prioritize basic needs, suppressing non-essential educational consumption.

#### 2.1.2. Other aspects of household resource allocation and human capital accumulation.

Health investments are closely linked to educational outcomes. Nutritional interventions have been proven to enhance human capital effectively [11] confirmed that nutritional education optimizes the dietary structure of low-income families [12] found that high-quality breakfasts improve academic performance by boosting adaptive motivation, while the lack of animal protein restrains the development of children in low-income countries [13]. However, risky health behaviors are widespread. Energy drink consumption is significantly associated with academic stress and lack of family supervision [14]. Excessive recreational screen time directly impairs academic achievement [15], and the risk of cannabis use reflects the interplay of genetic and socioeconomic factors [16]. The uneven distribution of health resources further exacerbates inequality. In Chile, low-income groups have seriously inadequate fruit and vegetable intake [17]. Nutritional taboos among rural pregnant women hinder fetal development [18]. During COVID-19, American minorities faced greater food shortage risks [19], and school staff in New Jersey encountered a cutoff of mental health support resources [20].

In conclusion, family educational expenditure is a complex and multifaceted phenomenon involving various variables and interaction mechanisms. Understanding these factors is instrumental in crafting effective educational policies for advancing social equity. Therefore, future research should shift its focus to cross-cultural. This approach will facilitate the optimal allocation of educational resources globally and contribute to a more equitable and efficient educational landscape worldwide.

### 2.2. Research on residents’ credit behavior

#### 2.2.1. Family finances and credit behavior: Dynamic exploration of time-varying bidirectional causal relationships.

The interplay between household financial decisions and credit acquisition is crucial in contemporary economic research. This section systematically reviews the existing literature to explore how families utilize credit resources to manage their financial status and how these decisions evolve over time and in different contexts. Special attention is given to the time-varying bidirectional causal relationship between family educational expenditure and residents’ credit behavior, and the heterogeneous mechanisms that emerge in this process.

Household Credit Acquisition and Financial Management. [21] unveiled the temporal trends of liquidity constraints among American households, highlighting that technological advancements reduced the cost of credit production and enhanced credit accessibility for marginal borrowers [22] emphasized the importance of credit acquisition for business operations, particularly in developing countries like Costa Rica [23] focused on the participation of low-income households in microfinance projects in Uganda, demonstrating that repeated exposure to financial services significantly bolstered families’ investment capabilities in health, education, and consumption.

Dynamic Changes and Heterogeneity Mechanisms. The research by [24] and [25] compared the distinct insights derived from micro and macro data in the study of wealth accumulation and savings behavior, highlighting the unique perspectives offered by different types of data sources [26] further explored the division of financial responsibilities between spouses and its impact on credit knowledge and behavior, finding that higher-income partners are more likely to manage household finances [27] examined the effect of consumer credit on individual and household consumption behavior in China, identifying a positive correlation between consumer loans and spending while also noting urban-rural disparities.

Credit Behavior in the Digital Era. With the advent of the digital age [28], outlined the changes brought by modern digital processes to financial markets, particularly their impact on household credit behavior [29] investigated the impact of household financial behavior on financial system’s stability, aiming to identify the relationship between the two [30] emphasized the crucial role of financial literacy in shaping borrowing behavior and income levels among rural households in China, indicating that financial knowledge can effectively enhance credit access and improve household financial conditions.

Analysis of High-Frequency Data Post-COVID-19 Pandemic. Since the onset of the COVID-19 pandemic in 2020, the utilization of high-frequency data for near real-time analysis has emerged as a novel trend in the study of household finance and credit behavior. This burgeoning area of research examines the impact of financial education on credit behavior, particularly among young people, and often relies on panel data from credit reports. Significant contributions to household debt, especially student loan debt, and the cyclical nature of household and corporate credit in emerging markets remain persistent themes of investigation.

Since the onset of the COVID-19 pandemic in 2020, high-frequency data for near real-time analysis has emerged as a novel trend in the study of household finance and credit behavior [31]. Household debt is another critical area of study, including its overall default rates [32] and the significant contribution of student loan debt [33]. The cyclical nature of household and corporate credit in emerging markets remains a persistent investigation theme [34], with some studies emphasizing the role of mortgage debt in financial vulnerability [35].

The broad context of household finance includes personal financial management across various life stages, covering topics such as debt, credit, housing, transportation, and retirement planning [36]. Data on household financial inclusion collected through surveys, such as bank account ownership [37], is also a research subject in this field. The ever-evolving landscape of consumer payments and household finance necessitates ongoing research, including examining high-risk credit behaviors, particularly among college students, and the interplay of financial literacy and self-efficacy [38].

#### 2.2.2. Household education expenditure and residents’ credit behavior: Time-varying bidirectional causality and heterogeneous mechanisms.

Household education expenditure and residents’ credit behavior represent two pivotal domains that profoundly impact family well-being and financial stability. This section comprehensively reviews the extant literature to examine the intricate interplay between these two variables and their dynamic attributes. In particular, it emphasizes delving into the time-varying bidirectional causality and probing into the heterogeneous mechanisms at work.

The Impact of Cultural and Religious Values on Household Education Expenditure. [10] research highlights how culture and religious beliefs shape household consumption patterns. In Makassar City families, Islamic economic principles guide consumption decisions, prioritizing needs over desires. This cultural orientation influences daily consumption choices and may indirectly affect educational resource investment decisions, which indicates the significant role of religious teachings in shaping household consumption habits.

Regarding the driving factors of residents’ credit behavior, [39] utilized panel data analysis in China to reveal the driving factors of household borrowing behavior, which often deviate from classical economic theories. Their research demonstrates that various factors, including income levels, financial literacy, and the social security system, shape household debt levels. Comprehending these influencing factors holds significant value for policymakers and financial institutions in devising effective household debt management strategies [40] analyzed the credit behavior of rural residents engaged in income-generating projects, assessing the impacts of credit on educational opportunities, health, social activities, and economic outcomes such as income and consumption. The study highlighted the importance of credit programs in supporting rural households and enhancing their overall well-being while pointing out unequal credit access.

On the impact of external financial support on household financial behavior [41] examined how remittances influence household consumption and investment decisions in Province No. 5 of Nepal. Their study confirmed that external financial support, particularly international remittances, plays a significant role in shaping household financial behavior. The research revealed that such remittances are not merely a short-term source of funds but can also significantly influence long-term household financial planning and resource allocation strategies.

Credit behavior in the context of health and education [42] explored the impact of nutritional education interventions on breakfast consumption among preparatory school students in Egypt, applying Pender’s Health Promotion Model to evaluate how nutrition education influences students’ breakfast – consumption behavior [43] highlighted the importance of counseling in improving student motivation within higher education settings. These studies indicate that credit behavior in educational environments can indirectly enhance household well-being by improving health and educational quality.

#### 2.2.3. Credit behavior and financial resource allocation mechanisms.

Credit market participation significantly impacts household resource optimization. In terms of credit accessibility, while digital finance enhances participation, it also increases debt risks [44]. Social networks facilitate consumption through informal credit [45], and forest tenure reform strengthens the willingness to access formal credit [46]. There is a dynamic interaction between educational investment and credit. Inclusive finance increases educational investment by reducing borrowing costs [47], yet post-compulsory education expenses significantly crowd out illiquid asset allocation [48]. During crises, credit behavior exhibits new characteristics. High-frequency data from the COVID-19 period revealed household credit contraction [31], and student loan debt exacerbated financial vulnerability [35]. Notably, housing credit has become a tool for asset stratification. Highly-educated groups are more likely to utilize provident fund loans [49], and African Americans in the U.S. significantly lag in asset accumulation at the same debt level [50].

#### 2.2.4. Evaluation of social policy intervention effects.

Policy design directly impacts resource allocation efficiency. In educational equity policies, Indonesia’s targeted scholarships have improved poor students’ GPA by 0.3–0.5 points [51]. In contrast, the U.S. “education theft” law institutionalizes the exclusion of low-income groups through fraudulent residency requirements [52]. Health security policies have shown differentiated effects: India’s health insurance has narrowed the gender gap in girls’ education [53], Lebanon’s school feeding programs have improved the nutritional status of refugee children [54], and Israel’s lack of emergency meal plans has exacerbated food crises [55]. Adjustments in fertility policies have triggered a chain of reactions. The “one-child-two-child” policy has led to a 30% decrease in per capita educational expenditure [56], and the continuous global decline in fertility rates requires the restructuring of educational resource allocation [57]. Digital technology empowerment has shown potential, with digital payments alleviating income inequality by increasing the entrepreneurship rate by 12.7% [58] and Brazil’s mobile medical teams optimizing resource efficiency in remote areas [59].

#### 2.2.5. Intergenerational transmission and pathways of inequality reproduction.

Intergenerational resource transmission forms a mechanism for the solidification of inequality. Regarding educational transmission across generations, grandparents’ education affects grandchildren via parents as intermediaries [60,61], and son preference weakens the transmission effect, while in families with multiple children, girls’ access to resources is diluted [2]. [62] emphasize that socioeconomic status, parental involvement, and family environment shape differences in academic achievement. Asset stratification unfolds in spatial dimensions. In South Korea, the difference in extracurricular education time between urban and rural areas is as high as twice [63]. In Nepal, hydropower immigrants rely on physical assets to restore their livelihoods [64]. Gender division of labor continues to affect resource allocation. In mixed-gender families, bill payment is determined by income level, but women still mainly undertake shopping responsibilities [65].

Despite these insights, three limitations persist: (1) predominant use of static models ill-suited for capturing bidirectional causality; (2) inadequate quantification of heterogeneity mechanisms; (3) omission of time-varying policy effects. Our study aims to resolve these gaps.

### 2.3. Research gaps and potential contributions

This study bridges critical gaps in understanding the dynamic relationship between household education expenditure and resident credit behavior. Prior research predominantly employed static or unidirectional frameworks, overlooking time-varying bidirectional causality and heterogeneous mechanisms across socioeconomic strata. Our work addresses these limitations through four integrated innovations.

Methodologically, we introduce a novel dual-model framework combining TVP-SV-VAR (Time-Varying Parameter Vector Autoregression with Stochastic Volatility) and QVAR-DY (Quantile Vector Autoregression with Diebold-Yilmaz spillover) to capture evolving dynamics and heterogeneity. The TVP-SV-VAR model uncovers time-varying causal relationships and impulse responses across economic cycles, enabling the first systematic analysis of how bidirectionality shifts amid economic uncertainty. Complementarily, the QVAR-DY framework examines asymmetric spillover effects across quantiles (e.g., low/medium/high risk), revealing fundamental differences in how household risk profiles shape the education-credit nexus. Together, these models overcome traditional static limitations: TVP-SV-VAR tests dynamic causality (Hypothesis H1) and moderating mechanisms (H3), while QVAR-DY validates quantile heterogeneity (H2) and spillover dynamics (H4).

Beyond methodology, we establish a comprehensive theoretical framework addressing heterogeneity often neglected in existing literature. Through dynamic quantile analysis, we identify divergent patterns in educational investment and credit decision-making among households stratified by socioeconomic status, education levels, and credit needs. Critically, we incorporate household characteristics like financial literacy and income stability as key moderators a theoretical advancement enabling granular analysis of how these attributes shape responses under economic uncertainty.

Finally, our approach revolutionizes policy evaluation. Moving beyond static assessments, we employ time-varying parameter modeling to track policy effect evolution – including time lags, variability, and persistence across economic environments and policy cycles. This dynamic assessment provides a scientific basis for designing adaptive policies that account for long-term consequences and regime shifts. Collectively, these contributions resolve literature gaps in: 1) dynamic bidirectional modeling, 2) socioeconomic heterogeneity, 3) financial literacy mediation, and 4) static policy evaluation–offering unprecedented insights into household financial behavior.

### 2.4. Research hypotheses

Based on an in-depth analysis of historical literature, prior studies have extensively examined family educational consumption and household lending behavior separately. However, research on their time-varying two-way causal relationship and underlying heterogeneous mechanisms remains limited. To address this gap, this study proposes a set of hypotheses to systematically investigate the dynamic interplay between educational expenditure and credit behavior.

H1: Dynamic two-way causal relationship

A time-varying two-way causal relationship exists between family educational consumption and household lending behavior. The strength of this relationship fluctuates countercyclically with economic policy uncertainty. When economic uncertainty exceeds a certain threshold, the promoting effect of educational consumption on credit demand decelerates more rapidly.

H2: Quantile heterogeneity

In low-risk households, educational consumption has a higher spillover effect on lending behavior, reflecting a dominant “education investment-driven credit demand” relationship. In high-risk households, lending behavior is more sensitive to educational consumption, but high debt leverage weakens the capacity for educational expenditure.

H3: Heterogeneous moderating mechanisms

The higher a household’s disposable education income, the stronger the pulling effect of educational consumption on lending behavior. Rising credit interest rates inhibit the promotion of credit in educational consumption. As economic policy uncertainty intensifies, households reduce credit-supported educational consumption.

The higher a household’s disposable education income, the stronger the pulling effect of educational consumption on lending behavior. Rising credit interest rates inhibit the promotion of credit in educational consumption. Furthermore, households with higher financial literacy demonstrate greater resilience in maintaining education expenditure during economic uncertainty. As economic policy uncertainty intensifies, households with low financial literacy reduce credit-supported educational consumption more significantly.

H4: Multi-period spillover effects

The short-term impact of educational consumption on the credit market is significantly stronger than its medium-to-long-term feedback effect on educational consumption itself, with a policy implementation lag of approximately one year.

## 3. Research methods

### 3.1. Model construction

#### 3.1.1. Construction of the TVP-SV-VAR model.

The TVP-SV-VAR model (Time-Varying Parameter Vector Autoregression with Stochastic Volatility) has been extensively utilized in recent years to study the dynamic effects of macroeconomic policy interventions. Compared to traditional VAR models, the TVP-SV-VAR model allows for evolving coefficients and variances, thus capturing the potentially non-linear structural relationships among variables. Given that the interactions between fiscal, monetary policies, and their combined effects on economic fluctuations are not static, this model can effectively illustrate the dynamic spillover effects from U.S. macroeconomic policy adjustments on China’s economy, showcasing its superiority.

A conventional VAR model is typically formulated as:


$$y_{t}=F_{1}y_{t-1}+\cdots +F_{s}y_{t-s}+u_{t},\;t=s+1,\ldots ,n$$ (1)

Where, $y_{t}$ is a k×1 vector, $F_{1},F_{2},\ldots ,F_{s}$ are k×k coefficient matrices; and $u_{t}$ represents a k×1 structural shock term. Assuming $u_{t}\sim N(0,\Sigma )$, where Σ is a lower triangular matrix representing the contemporaneous structure among variables.


$$\Sigma =[\begin{array}{cccc} \sigma_{1} & 0 & \cdots & 0\\ 0 & \sigma_{2} & \cdots & 0\\ \vdots & \vdots & \ddots & \vdots \\ 0 & 0 & \cdots & \sigma_{k} \end{array}]$$ (2)

$$A=[\begin{array}{cccc} 1 & 0 & \cdots & 0\\ a_{21} & 1 & \cdots & 0\\ \vdots & \vdots & \ddots & \vdots \\ a_{k1} & a_{k2} & \cdots & 1 \end{array}]$$ (3)

Letting $B_{i}=A^{-1}F_{i}(i=1,\ldots ,s)$ and multiplying both sides of equation (1) by $A^{-1}$, we obtain:


$$y_{t}=B_{1}y_{t-1}+\cdots +B_{s}y_{t-s}+A^{-1}\Sigma e_{t},\;e_{t}\sim N\left(0,I_{k}\right)$$ (4)

Following Primiceri(2005), if we define $X_{t}={\left[y_{t}^{\prime },y_{t-1}^{\prime },\ldots ,y_{t-s}^{\prime }\right]}^{\prime }$ and stack the rows of matrices $B_{i}$ into a vector β, equation (4) can be transformed into: $y_{t}=X_{t}^{\prime }\beta +A^{-1}\Sigma e_{t}$

Incorporating time-variation yields the TVP-SV-VAR model:


$$y_{t}=X_{t}^{\prime }\beta_{t}+A_{t}^{-1}\Sigma_{t}e_{t},\;e_{t}\sim N\left(0,I_{k}\right),\;t=s+1,\ldots ,n$$ (5)

In equation (5), parameters $\beta_{t},A_{t}$,and $\Sigma_{t}$ all vary over time.Furthermore, let $\alpha_{t}$ denote the stacked vector of the lower triangular elements of matrix $A_{t,}$ and $h_{t}={\left(h_{t1},\ldots ,h_{tk}\right)}^{\prime }$ represent the log volatility matrix {SV}. For all j= 1,…,k and $t=s+1,\ldots ,n,h_{jt}=ln\sigma_{jj}^{2}$. All parameters in equation (5) follow a first-order random walk process:


$$\beta_{t+1}=\beta_{t}+\mu_{\beta_{t}}$$

$$\alpha_{t+1}=\alpha_{t}+\mu_{\alpha_{t}}$$ (6)

$$h_{t+1}=h_{t}+\mu_{h_{t}}$$

$$(\begin{array}{c} {\mu_{\beta }}_{t}\\ \mu_{\alpha_{t}}\\ \mu_{h_{t}} \end{array})\sim N\left((\begin{array}{c} 0\\ 0\\ 0 \end{array}),[\begin{array}{ccc} \Sigma_{\beta } & 0 & 0\\ 0 & \Sigma_{\alpha } & 0\\ 0 & 0 & \Sigma_{h} \end{array}]\right)$$ (7)

Stochastic Volatility Specification and Parameter Rationale. The random walk assumption in equation (6) follows the seminal framework of [66] and [67], where the innovation terms $\mu_{\beta_{t}}$, $\mu_{\alpha_{t}}$, and $\mu_{h_{t}}$ are assumed to be independently distributed as equation (7).

This Gaussian distribution assumption serves two critical purposes in capturing financial dynamics:

Time-Varying Persistence: Allows coefficients to evolve gradually, reflecting households’ adaptive financial decision-making under changing economic conditions [68].

Volatility Clustering: The log-volatility process $h_{t}$ captures heteroskedasticity commonly observed in credit behavior data during crisis periods (e.g., COVID-19 shocks in 2020–2022).

Hyperparameter Selection: The covariance matrices Σβ, Σα, Σh are diagonal as per Nakajima (2011)’s recommendation, implying independence between parameter innovations. This specification avoids overparameterization while maintaining flexibility.

Estimation Method. To ensure the robustness and accuracy of the parameter estimates, we employ the Markov Chain Monte Carlo (MCMC) sampling method, as proposed by [67]. This approach allows us to estimate the time-varying parameters more accurately by accounting for the stochastic nature of the volatility and the evolving coefficients. Specifically, we assume that the shocks to the time-varying parameters are uncorrelated and that the covariance matrices $\Sigma_{\beta },\Sigma_{\alpha \iota }$ and $\Sigma_{h}$ are diagonal matrices. This assumption simplifies the estimation process and enhances the reliability of the results. By adopting these advanced methods, we aim to provide a comprehensive and accurate analysis of the dynamic relationships between household education expenditure and credit behavior, contributing to a deeper understanding of the underlying mechanisms and policy implications.

These formulations and descriptions provide a foundational framework for the TVP-SV-VAR model, facilitating an in-depth analysis of the time-varying bidirectional causality between household education expenditure and credit behavior.

#### 3.1.2. Quantile vector autoregression with diebold-yilmaz spillover framework (QVAR-DY).

The QVAR-DY model integrates the Quantile Vector Autoregression (QVAR) framework with the Diebold-Yilmaz (DY) spillover index methodology, which is an advanced econometric tool designed to capture the heterogeneous spillover effects under different market conditions. Unlike traditional models based on conditional means, the QVAR-DY framework can effectively address the limitations of underestimating the impact of extreme events, especially during periods of high market volatility when inter-market connectivity significantly increases. This model provides a more accurate representation of risk spillovers by fitting the data at different conditional quantiles and revealing the tail risk contagion characteristics between markets under extreme upswings and downturns.

(1) Model formulation

The QVAR model extends traditional VAR by incorporating conditional quantiles, allowing estimation of asymmetric spillovers. The QVAR(p) system at quantile level τ is:


$$\begin{array}{c} x_{t}(\tau )=\mu (\tau )+\sum {\mathrm{\backslash nolimits}}_{i=1}^{p}\Phi_{i}(\tau )x_{t-i}(\tau )+u_{t}(\tau ) \end{array}$$ (8)

where:

$x_{t}(\tau )$ is an N×1 vector of endogenous variables at conditional quantile τ.

μ(τ) is an N×1 vector of conditional means.

$\Phi_{i}(\tau )$ is an N×N matrix of coefficients representing the impact of lagged terms on the current values.

$u_{t}(\tau )$ is error term with zero τ-quantile conditional mean.

p is the number of lags, determined by model selection criteria such as the Schwarz Information Criterion (SIC).

(2) Spillover measurement via DY framework

Step 1: QVMA Representation

Generalized Forecast Error Variance Decomposition. The conversion from QVAR to QVMA representation draws on the fundamental principle of impulse response analysis in time series econometrics. This transformation allows us to quantify how a unit shock to one variable propagates through the system over time. The generalized forecast error variance decomposition method is employed to quantify the spillover effects between different variables. The key steps are outlined below:

Transforming the QVAR Model into an Infinite-Order Quantile Vector Moving Average (QVMA) Process:


$$\begin{array}{c} x_{t}(\tau )=\mu (\tau )+\sum {\mathrm{\backslash nolimits}}_{j=0}^{\infty }\Psi_{j}(\tau )u_{t-j}(\tau ) \end{array}$$ (9)

where $\Psi_{j}(\tau )$ captures the cumulative effect of shocks at lag j.

Step 2: Generalized Variance Decomposition

For forecast horizon H, the contribution of variable k to variable i’s forecast error variance at τ is:


$$\theta_{ik}(H,\tau )=\frac{\sum_{h=0}^{H-1}{\left(e_{i}^{⊤}\Psi_{h}(\tau )\Sigma_{T}e_{k}\right)}^{2}}{\sum_{h=0}^{H-1}e_{i}^{⊤}\Psi_{h}(\tau )\Sigma_{T}\Psi_{h}^{⊤}(\tau )e_{i}}$$ (10)

$\Sigma_{T}$: Variance-covariance matrix of $u_{t}(\tau )$.

$e_{i}$: Selection vector with 1 at position i and 0 elsewhere.

Step 3: Standardized Spillover Index

Normalization. To ensure comparability of the spillover indices, $\theta_{ik}(h,\tau )$ is normalized to obtain the standardized spillover index $\theta_{ij}(H,\tau ):$


$$\theta_{ij}(H,\tau )=\frac{\theta_{ij}(h,\tau )}{\sum_{k=1}^{N}\theta_{ik}(h,\tau )}$$ (11)

Here the indicator measures the extent to which variable k contributes to the variance of the prediction error of variable i.

(3) Directional and net spillover indices

Total Spillover Index(TO). Represents the total spillover effect of variable i to other variables


$$\begin{array}{c} TO_{i}(H,\tau )=\sum {\mathrm{\backslash nolimits}}_{j\neq i}\theta_{ij}(H,\tau ) \end{array}$$ (12)

Spillover-in Index (FROM). Represents the total spillover effect received by variable i from other variables:


$$\begin{array}{c} FROM_{i}(H,\tau )=\sum {\mathrm{\backslash nolimits}}_{j\neq i}\theta_{ji}(H,\tau ) \end{array}$$ (13)

Net Spillover Index (NET). Indicates the net spillover effect of variable i, calculated as the difference between total spillover and spillover-in:


$$NET_{i}(H,\tau )=TO_{i}(H,\tau )-FROM_{i}(H,\tau )$$ (14)

Total Connectivity Index (TCI). Reflects the overall spillover level within the network


$$\begin{array}{c} TCI(H,\tau )=\frac{1}{N}\sum {\mathrm{\backslash nolimits}}_{i=1}^{N}{TO}_{i}(H,\tau ) \end{array}$$ (15)

(4) Advantages of QVAR-DY

Tail risk sensitivity. Captures asymmetric spillovers during extreme market movements (e.g., crises).

Dynamic analysis. TCI and NET indices reveal time-varying contagion patterns across quantiles.

Policy relevance. Informs regulators on systemic risk sources under stress scenarios.

Estimation. Use quantile regression to estimate Φi(τ) and bootstrap methods for inference.

This study constructs a TVP-SV-VAR model and a QVAR-DY model to validate hypotheses H1–H4, which pertain to the time-varying two-way causal relationship between family educational consumption and household lending behavior and the moderating role of economic uncertainty. Empirical analysis uses data from urban Chinese households from January 2015 to December 2024.

### 3.2. Data sources and variable definitions

The data utilized in this study spans from January 2015 to December 2024, ensuring both the timeliness and representativeness of the dataset. This comprehensive time frame allows for a thorough examination of the dynamics in family behavior over a decade, capturing both short-term fluctuations and long-term trends. Our dataset encompasses both the demand side and supply side of family behavior. On the demand side, we focus on key factors such as income and education consumption, which are crucial in shaping household consumption and investment decisions. On the supply side, we incorporate important determinants like interest rates and credit availability, significantly influencing the feasibility and attractiveness of various financial choices for families. In addition to these fundamental variables, we integrate macroeconomic risks and debt constraints into our analysis by including Economic Policy Uncertainty (EPU) and household debt-to-income ratio. These indicators provide a broader context for understanding how macroeconomic conditions and financial stability affect family behavior. The specific variables included in our dataset are as follows:

Educational Disposable Consumption (EduC). Household income allocated is to educational expenses, as well as real educational income.

Educational Disposable Income (EduI). Income is available for educational purposes, real educational spending.

Loan Index (LoanI). The year-on-year growth rate of loans, calculated to eliminate the influence of seasonal factors.

SHIBOR (SHIB). Shanghai Interbank Offered Rate, reflecting borrowing costs in the financial market.

Consumer Confidence (ConsC). Households’ optimism or pessimism about the economy and their financial situation.

Economic Policy Uncertainty (EPU). Level of uncertainty in economic policies.

Household Debt-to-Income Ratio (HhDL). Debt burden carried by households.

All data are sourced from the China Statistical Yearbook, CSMAR, and Wind clients and the China EPU index developed by [69]. By incorporating these variables, our study aims to comprehensively understand of the factors influencing family behavior from both macroeconomic and microeconomic perspectives.

### 3.3. Descriptive statistical analysis and BDS test analysis

(1) Descriptive statistical analysis

This study is based on 120 monthly observations from January 2015 to December 2024. Through descriptive statistics, it reveals the dynamic characteristics and economic connotations of the core variables (Table 1). Educational consumption expenditure (EduC) exhibits considerable volatility (standard deviation of 14.78), and its value range of [−44.26, 49.85] shows a clear bimodal distribution trend. Negative sample points indicate that some families may maintain rigid educational expenditure by incurring debt or cutting necessary consumption [70], while extreme positive values reflect the growing scale of educational investment by high-income groups. This “K-shaped divergence” phenomenon aligns closely with the intergenerational transmission mechanism of educational returns [71]. The distribution characteristics of disposable income for education (EduI) (standard deviation of 27.40) further reinforce this conclusion. Its asymmetric relationship with EduC implies that policy shocks such as the “double reduction” policy have heterogeneous impacts on family behavior [72].

**Table 1:** Descriptive statistics.

Variable	Obs	Mean	Std. dev.	Min	Max
SHIB	120	2.749154	0.8161886	1.4155	5.040905
EduC	120	4.786955	14.78479	−44.26021	49.84843
LoanI	120	17.75663	23.96841	−11.50793	102.9021
ConsC	120	−0.0136725	1.010476	−1.612668	1.267858
EduI	120	47.08128	27.40474	−44.17193	134.7252
EPU	120	259.5597	118.8559	63.93907	649.0725
HhDL	120	6.204478	1.12218	4.080235	8.016461

Supply-side variables reveal the policy-oriented cyclicality of financial markets. Loan growth rate (LoanI) shows extreme volatility (standard deviation of 23.97, range of [−11.51, 102.90]). It captures the structural breakpoints in China’s credit cycle. The credit contraction caused by the 2017 deleveraging policy and the credit expansion due to quantitative easing after the 2020 pandemic form a striking contrast [73]. The Shanghai Interbank Offered Rate (SHIB) maintains a mean of 2.75. However, its high standard deviation of 0.82 and extreme range of 4.08 indicate policy transmission overreaction in the money market [74]. This provides a micro-foundation for understanding household financing constraints.

Macroeconomic risk indicators display significant tail-risk features. The parameter characteristics of the Economic Policy Uncertainty Index (EPU) confirm the “uncertainty clustering effect” proposed by [69]. The extreme point in the fourth quarter of 2022 (649 points) accurately captures the compounded impact of the Russia-Ukraine conflict and the adjustment of COVID-19 prevention and control policies. The statistical characteristics of the household debt-to-income ratio (HhDL) are more policy-worrying significant. It means 6.20 times has exceeded the OECD safety threshold (5 times). Also, 62 samples surpass this critical value. The extreme value of 8.02 times indicates that some families are in a debt sustainability crisis [75]. This finding, together with the persistently low Consumer Confidence Index (ConsC) (mean of – 0.01), forms an explanatory closed loop. Under the joint effect of high uncertainty and debt constraints, the marginal propensity to consume of households is systematically suppressed.

The dynamic distribution and structural breakpoints of the above data, especially the extreme-value clusters of EduC and LoanI, indicate that conventional linear causal models may severely underestimate the time-varying interaction between educational expenditure and credit behavior. This necessitates a dynamic quantile framework to capture the phase-specific evolution of variables.

(2) BDS test analysis

Table 2 presents the BDS test statistics for variables across different dimensions, with varying significance. SHIB, EPU, LoanI, EduI, and ConsC exhibit significant nonlinear relationships in various dimensions, suggesting their association with complex patterns that linear models cannot fully capture. Additionally, EduC is significant in M2, M4, and M6 dimensions; HhDL in M2, M5, and M6; EduI in M2 and M4-M6; and CONSC in M5 and M6. These results further indicate the presence of widespread nonlinear characteristics among these variables, suggesting that traditional linear models may not be able to uncover their underlying relationships adequately.

**Table 2:** BDS test.

Dimension	EduC	SHIB	HhDL	LoanI	EPU	EduI	ConsC
M2	0.0062***	0***	0.0062***	0***	0***	0.0528***	0***
M3	0.6093	0***	0.3137	0***	0.0002***	0.1269	0***
M4	0.0978***	0***	0.2396	0***	0.0001***	0.0038***	0***
M5	0.3838	0***	0.0217***	0***	0.0001***	0.0018***	0***
M6	0.0646***	0***	0.0093***	0***	0.0002***	0.001***	0***

Note: The values in Table 2 represent p-values.

### 3.4. Data stationarity and lag order testing

Table 3 presents the results of the Augmented Dickey-Fuller (ADF) test for unit roots across different variables, assessing the stationarity of the time-series data. These results indicate that most variables achieve stationarity after first-order differencing, providing a foundation for subsequent time-series analysis. Table 4 outlines the determination of the lag order.

**Table 3:** Unit root test results.

Variable	Type	ADF_Statistic	p_Value	Stationary
SHIB	Original	−2.53417	0.35471786	No
SHIB	First Difference	−5.808542	0.01	Yes
EduC	Original	−4.178118	0.01	Yes
LoanI	Original	−3.748085	0.02387518	Yes
LoanI	First Difference	−3.365463	0.06368958	No
ConsC	Original	−1.495989	0.78590081	No
ConsC	First Difference	−5.026902	0.01	Yes
EduI	Original	−4.880426	0.01	Yes
EPU	Original	−2.786148	0.2500647	No
EPU	First Difference	−6.219564	0.01	Yes
HhDL	Original	−2.995801	0.16299042	No
HhDL	First Difference	−6.17418	0.01	Yes

**Table 4:** Lag order test results.

Lag Order	AIC(n)	HQ(n)	SC(n)	FPE(n)
1	3.41E + 01	3.495363e + 01*	3.622989e + 01*	6.36E + 14
2	3.37E + 01	3.53E + 01	3.78E + 01	4.34E + 14
3	3.311380e + 01*	3.56E + 01	3.91E + 01	2.631171e + 14*
4	3.35E + 01	3.68E + 01	4.15E + 01	4.54E + 14

## 4. Data analysis and exploration of causal mechanisms

### 4.1. Granger causality test: From linear to time-varying theoretical exploration

#### 4.1.1. Linear granger causality test: Fundamental relationships between household educational consumption and credit behavior.

**Fig 1:**
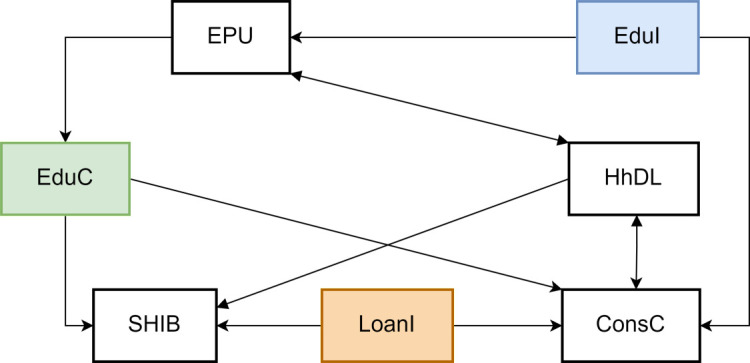
Linear granger causality test. **NOTE:** Fig 1 is based on a linear Granger causality test.

This study exhibits the interplay among educational consumption (EduC), loan availability (LoanI), and consumer confidence (ConsC) through Fig 1, unveiling the core dynamics of household financial behavior. It particularly emphasizes educational consumption as a vital component of household expenditure, whose decisions are profoundly influenced by expectations of future income. Amid heightened economic policy uncertainty (EPU), households tend to evaluate the returns on educational investments more cautiously. This uncertainty indirectly moderates educational consumption decisions by affecting expected benefits.

Credit availability plays a pivotal role in this process. Relaxed lending conditions enhance household liquidity and consumption capacity, fostering an increase in educational expenditures, especially when the anticipated returns on educational investments are expected to lead to higher future incomes. Conversely, a tight credit environment constrains households’ borrowing capacity and willingness, inhibiting educational consumption.

Furthermore, fluctuations in consumer confidence also significantly impact educational consumption. During of declining confidence, households may reduce discretionary spending, including education, to manage potential financial risks. Therefore, the interplay among educational consumption, credit availability, and consumer confidence reflects how households optimize their financial resources under different economic conditions and underscore the central role of educational investments in households’ long-term financial planning.

#### 4.1.2. Time-varying granger causality test: The dynamic interaction between household educational consumption and credit.

**Fig 2:**
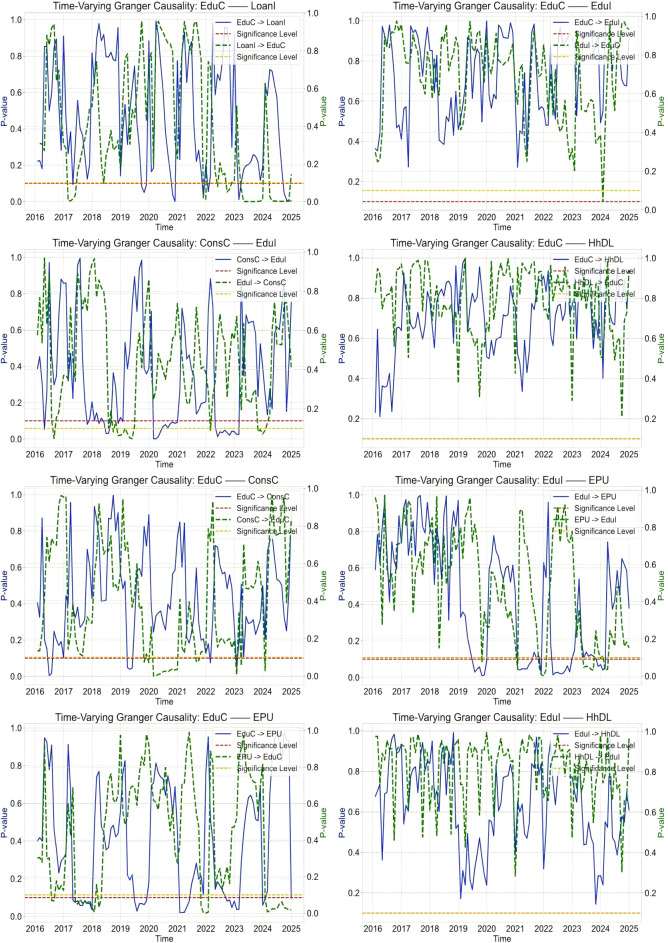
Time-varying granger causality test. **NOTE:** Fig 2 displays only a portion of the time-varying causality diagram; please refer to Appendix Fig 1 the complete view of the remaining related diagrams.

Building upon the previous exploration of linear Granger causality, we have identified static relationships among various economic variables. However, given the complexity and dynamism of economic relationships in the real world, it is particularly necessary to adopt a time-varying Granger causality test further to gain deeper insights into the evolving interactions, as illustrated in Fig 2.

Causal Relationship Between Consumption and Educational Expenditure. From 2016 to 2025, the causal impact of consumption on educational expenditure underwent significant changes. Although this causal relationship was significant in the early years (2016–2017), it exhibited considerable volatility. Subsequently (2018–2020), this causal link became more stable, indicating that changes in consumption had a strong predictive power for educational expenditure. However, in recent years (2021–2025), although the causal relationship persists, its volatility has increased, which may be related to changes in the macroeconomic environment.

Causal Relationship Between Educational Expenditure and Economic Policy Uncertainty. The study finds that the causal relationship between educational expenditure and economic policy uncertainty exhibited phased characteristics from 2016 to 2024. Initially (2015–2017), there was a significant causal link between the two; in the medium term (2018–2020), this relationship further strengthened, reflecting an increased importance of educational expenditure in explaining economic policy uncertainty. However, in the later period (2021–2024), the causal stability between them declined due to intensified economic fluctuations, adding complexity to the analysis.

The causal relationship between educational expenditure and loans also exhibited distinct phase characteristics during the study period (20165–2025). In the early (2015–2017) and medium (2018–2020) terms, a relatively significant and increasingly strong causal relationship was observed, indicating the supportive role of loans in educational expenditure and the driving effect of educational expenditure on loan demand. However, recently (2021–2024), with adjustments to financial policies and uncertainties in the economic situation, the volatility of this causal relationship has increased, demonstrating a certain degree of instability.

The causal relationship between educational expenditure and household debt gradually intensified from an initial significant state to the medium term between 2015 and 2024, reflecting the importance of household debt in supporting educational expenditure and its reverse impact. However, in the later period, this causal relationship became more complex due to changes in the economic environment, with correspondingly increased volatility.

The causal analysis between education loans and economic policy uncertainty reveals that their relationship evolved from significant to enhanced and became more complex due to economic fluctuations between 2015 and 2024. Especially between 2018 and 2020, the explanatory power of education loans for economic policy uncertainty peaked. However, subsequently, due to the increase in economic instability, the stability of this causal relationship declined.

Economic Drivers of Dynamic Causality Shifts. Major policy shifts and economic events drive the observed fluctuations in causality strength: During 2015–2017, interest rate liberalization reforms enhanced credit accessibility (PBOC 2015), strengthening the EduC→LoanI linkage, while the “Double First-Class” initiative boosted higher education investment. From 2018 to 2020, US-China trade tensions (EPU index peak 382 in Q3 2019) increased households’ reliance on education credit for hedging, though COVID-19 loan moratoriums temporarily decoupled debt from education spending. Post-2021, the “Double Reduction” policy abruptly reduced supplementary education demand, weakening the causal chain, while the property market crisis (e.g., Evergrande defaults) triggered credit contraction that disproportionately affected education loans. Based on the above analysis, Hypothesis 1 is confirmed.

**Fig 3:**
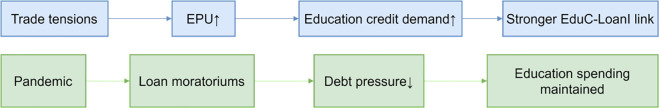
Key transmission mechanisms.

The key transmission mechanisms can be summarized into two transmission mechanisms, as illustrated in Fig 3.

### 4.2. TVP-SV-VAR analysis of household education expenditure and residents’ credit behavior

**Fig 4:**
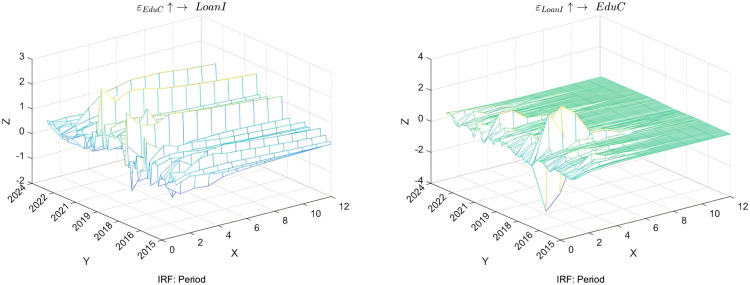
Time-varying impulse response function of education expenditure and credit. **NOTE:** The X-axis (Period) represents the time periods. The Y-axis represents the years. The Z-axis represents the values of the impulse response function.

This study aims to explore the time-varying bidirectional causality between household education expenditure and residents’ credit behavior, with a particular focus on education discretionary consumption (EduC) and loan indicators (LoanI), as shown in Fig 4. The research reveals that after a positive shock in household education expenditure, families initially tend to meet their funding requirements by increasing borrowing, leading to a significant rise in loan demand. However, as families identify alternative methods for budgetary balance, such as income growth or savings adjustments, this trend gradually diminishes, exemplifying the flexibility of household financial management strategies.

Further analysis underscores the significance of changes in loan conditions on household education expenditure. Enhanced loan accessibility or reduced costs can swiftly stimulate growth in household education investment, particularly in scenarios where educational resources are scarce or future returns are anticipated to be high. However, as debt accumulates, households reassess their decisions regarding education expenditure, leading to a deceleration and stabilization of growth in education consumption. Notably, households with varying income levels and socioeconomic backgrounds exhibit different sensitivities to changes in loan conditions, suggesting that policy design must consider these heterogeneous factors to ensure equitable access to educational resources. This analysis serves to validate Hypothesis 3.

Moreover, this study observes a notable impact of education expenditure on the credit market, primarily due to the involvement of substantial financial outlays in education consumption, which often necessitates credit support. This characteristic causes fluctuations in credit demand stemming from education consumption, thereby influencing the credit market. From a theoretical perspective, by integrating the life-cycle hypothesis and liquidity constraint theory, this study provides an in-depth analysis of the dynamic relationship between household education expenditure and residents’ credit behavior. According to the life-cycle hypothesis, households allocate resources intertemporally based on current and expected income to meet future needs. In contrast, the liquidity constraint theory emphasizes that improvements in the credit market environment offer opportunities for households to overcome liquidity barriers and increase investments in education.

**Fig 5:**
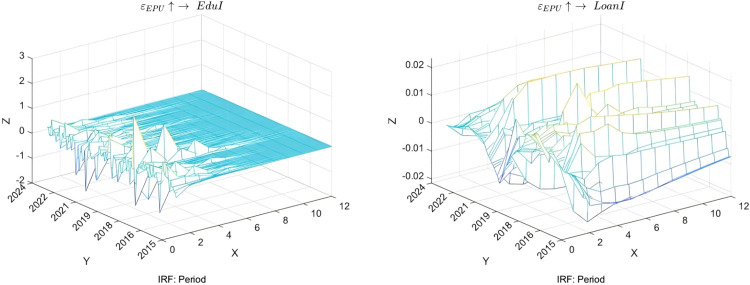
Time-varying impulse response of economic uncertainty, education expenditure, and credit.

This study adopts a dynamic perspective to examine the impact of economic uncertainty on household education investment and residents’ credit behavior, as illustrated in Fig 5. Regarding education disposable income (EduI), the research reveals that households tend to reduce education expenditure in the initial stages of heightened economic uncertainty to cope with potential financial pressures. This aligns with the expectations of the precautionary saving theory, which prioritizes basic living expenses over long-term investments. However, this impact gradually diminishes over time, suggesting that households can continue supporting their children’s educational needs by adjusting other expenditures or seeking additional income sources. This underscores the flexibility of household financial management strategies, and the importance of social safety nets and policy interventions. This analysis serves to validate Hypothesis 3.

Regarding the variations in loan indicators (LoanI), the study finds that economic uncertainty also significantly affects households’ ability and willingness to access credit. As market risks escalate, financial institutions generally tighten credit standards in the short term, rendering the borrowing environment more stringent and restraining the growth of loan demand, particularly for households that rely on credit for education investment. In the medium term, with market adaptability and policy regulations taking effect, loan indicators demonstrate an inevitable recovery trend, indicating that households actively seek ways to maintain necessary consumption levels even during economic instability. In the long run, if economic uncertainty persists, households may develop a more cautious lending attitude, which will have profound implications for the credit market.

**Fig 6:**
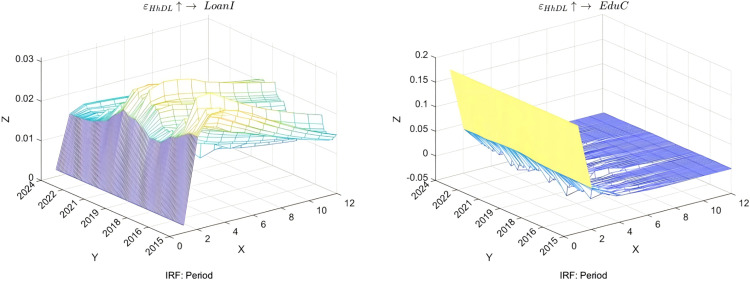
Impulse Response Function (IRF) of household leverage, education expenditure, and credit period. **NOTE:** The time-varying impulse response analysis conducted in this study among various economic indicators solely presents the interactive relationships among a few key economic indicators. For the time-varying impulse responses among other economic indicators not presented here.

Analysis of the Impact of Household Debt Levels on Loan Indicators and Education Consumption. In examining the influence of household debt levels (HhDL) on loan indicators (LoanI) and education consumption (EduC), the dynamic changes triggered by economic uncertainty have a significant impact on both, as shown in Fig 6. Research shows that when HhDL experiences a positive shock, financial institutions, considering risk control, typically adopt a more prudent stance, directly leading to a significant decline in LoanI in the short term. This phenomenon reflects the high sensitivity of credit suppliers to risk under market mechanisms. Over time, if the macroeconomic environment stabilizes and HhDL eases, the tension in the loan market will gradually improve, and LoanI will rebound accordingly. However, this recovery process is not instantaneous; instead, it accompanies complex adjustments such as policy interventions and rebuilding market confidence. This indicates that the effective implementation of macroeconomic policies plays a crucial role in stabilizing financial markets.

Furthermore, the impact of an elevated HhDL on EduC is equally significant. Under economic pressures, households prioritize fulfilling basic living needs, consequently reducing non-essential expenditures, such as investments in their children’s education. In the short term, the decrease in EduC reflects households’ immediate strategies to cope with financial distress; however, in the long run, this may inhibit the society’s overall human capital development. Notably, government support through financial subsidies, tax incentives, and other means can mitigate the adverse effects of high debt on households to a certain extent, aiding in maintaining necessary educational investments. Additionally, a well-established social security system plays a pivotal role in alleviating household financial burdens and fostering equitable distribution of educational resources.

### 4.3. Exploring the multidimensional causality between household education expenditure and residents’ credit behavior

In household economic decision-making, the significance of household education expenditure is increasingly prominent, exerting a notable influence on residents’ credit behavior. This study employs quantile regression to demonstrate the impact results at various quantile levels, analyzing the bidirectional causality and heterogeneity between household education expenditure and residents’ credit balances. Meanwhile, static, dynamic, and scale analyses are utilized to examine the spillover effects, with the calculation of overall and net spillover effects comprehensively exploring the multidimensional causality between the two.

#### 4.3.1. Assessment of static spillover effects.

**Fig 7:**
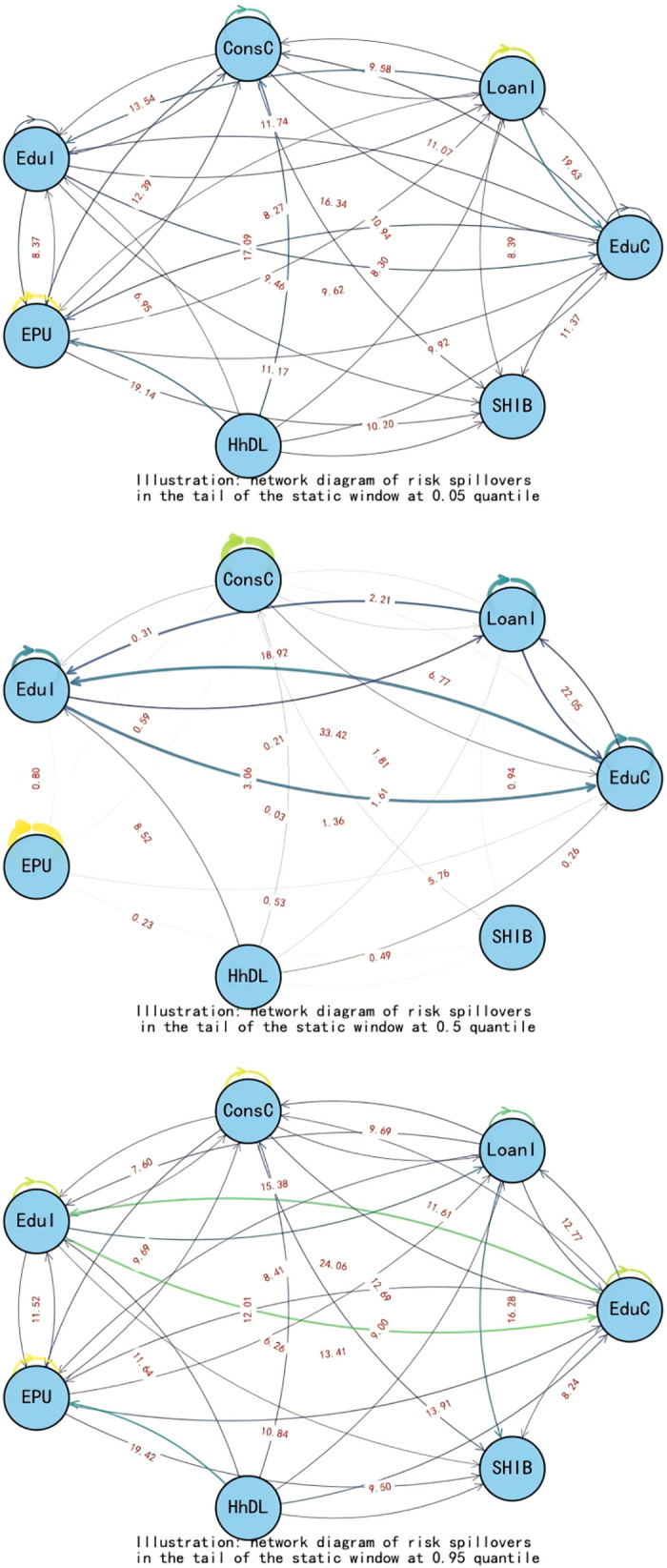
Static spillover effects.

When exploring the time-varying bidirectional causality between household education expenditure and residents’ credit behavior, we delve into the static spillover effects of tail risks across different quantiles (0.05, 0.5, 0.95). This analysis provides insights into the interactions among variables and their economic implications at various risk levels, as illustrated in Table 5 and Fig 7.

**Table 5:** Static spillovers.

Tail risk static spillover effect at the 0.05 quantile								
	SHIB	EduC	LoanI	ConsC	EduI	EPU	HhDL	FROM
SHIB	31.16	10.87	12.24	13.12	6.10	12.53	13.98	68.84
EduC	11.37	15.60	11.30	14.14	12.29	15.73	19.57	84.40
LoanI	8.39	19.63	34.67	8.80	17.29	6.56	4.66	65.33
ConsC	10.94	11.07	9.58	25.41	7.98	15.50	19.51	74.59
EduI	9.46	16.34	11.74	13.54	15.45	15.12	18.35	84.55
EPU	11.17	9.62	8.27	12.39	8.37	36.85	13.33	63.15
HhDL	10.20	9.92	8.30	17.09	6.95	19.14	28.41	71.59
TO	61.52	77.45	61.43	79.09	58.98	84.59	89.40	512.46
Inc.Own	92.68	93.05	96.10	104.49	74.43	121.44	117.80	cTCI/TCI
NET	−7.32	−6.95	−3.90	4.49	−25.57	21.44	17.80	85.41/73.21
NPT	2.00	2.00	4.00	3.00	1.00	5.00	4.00	
Tail risk static spillover effect at the 0.5 quantile								
	SHIB	EduC	LoanI	ConsC	EduI	EPU	HhDL	FROM
SHIB	94.35	1.88	0.44	1.29	0.99	0.34	0.72	5.65
EduC	0.26	44.14	16.57	0.75	35.00	0.10	3.19	55.86
LoanI	0.94	22.05	48.32	1.41	26.62	0.01	0.65	51.68
ConsC	1.81	6.77	2.21	81.90	3.53	0.63	3.14	18.10
EduI	0.03	33.42	18.92	0.31	42.60	0.35	4.38	57.40
EPU	0.53	1.36	0.21	0.59	0.80	94.25	2.25	5.75
HhDL	0.49	5.76	1.61	3.06	8.52	0.23	80.34	19.66
TO	4.06	71.23	39.97	7.41	75.45	1.65	14.34	214.12
Inc.Own	98.41	115.37	88.29	89.31	118.05	95.89	94.68	cTCI/TCI
NET	−1.59	15.37	−11.71	−10.69	18.05	−4.11	−5.32	35.69/30.59
NPT	3.00	5.00	3.00	0.00	6.00	1.00	3.00	
Tail risk static spillover effect at the 0.95 quantile								
	SHIB	EduC	LoanI	ConsC	EduI	EPU	HhDL	FROM
SHIB	23.96	12.19	17.25	10.84	10.05	13.52	12.18	76.04
EduC	8.24	27.06	13.74	8.94	23.67	10.58	7.76	72.94
LoanI	16.28	12.77	23.17	11.95	12.21	10.15	13.48	76.83
ConsC	12.69	11.61	9.69	28.72	8.50	12.88	15.91	71.28
EduI	6.26	24.06	15.38	7.60	27.72	10.02	8.95	72.28
EPU	10.84	13.41	8.41	9.69	11.52	29.85	16.28	70.15
HhDL	9.50	13.91	9.00	12.01	11.64	19.42	24.52	75.48
TO	63.80	87.95	73.47	61.02	77.60	76.57	74.57	514.99
Inc.Own	87.76	115.01	96.64	89.74	105.32	106.43	99.09	cTCI/TCI
NET	−12.24	15.01	−3.36	−10.26	5.32	6.43	−0.91	85.83/73.57
NPT	1.00	5.00	3.00	1.00	4.00	4.00	3.00	

At the low-risk level (0.05 quantile), in an economically stable environment, the spillover effects of education expenditure (EduC) and household debt-to-income ratio (HhDL) on other variables are significant, amounting to 84.40% and 71.59% respectively. This indicates that both play pivotal roles in risk propagation. The high spillover effect of education expenditure suggests that household education investment decisions are sensitive to changes in the economic environment. In contrast, the high spillover of household debt-to-income ratio demonstrates its impact on other economic variables. Additionally, economic policy uncertainty (EPU) accounts for 63.15% of the risk propagation to other variables, implying that even during periods of relative economic stability, policy uncertainty can still significantly influence household economic decisions, subsequently affecting the entire economic system.

At the medium-risk level (0.5 quantiles), as risk escalates to a moderate level, loan indicators (LoanI) and consumer confidence (ConsC) become the primary risk recipients, with only 51.68% and 18.10% of their risks spilling over to other variables respectively. The spillover effects of education expenditure (EduC) and education income (EduI) intensify, particularly with EduI’s risk spillover to other variables reaching 57.40%, indicating that education income has a more pronounced impact on the economic system under medium-risk conditions. An increase in education income fosters household consumption and investment and stimulates growth in credit demand, thereby playing a more significant role in economic activities.

At the high-risk level (0.95 quantiles), in states of extreme risk, the spillover effect of the household debt-to-income ratio (HhDL) further increases to 75.48%, indicating that under high-risk scenarios, the impact of household debt on other economic variables is particularly significant. Elevated household debt levels exacerbate volatility in financial markets, affecting credit conditions and household economic stability. Education income (EduI) and education expenditure (EduC) also exhibit high spillover effects at this risk level, reflecting the close link between education-related activities and household debt. Amid increased economic uncertainty, households become more reliant on education income to sustain their economic situations, while education expenditure influences other economic variables through changes in credit demand.

#### 4.3.2. Analysis of dynamic spillover effects.

**Fig 8:**
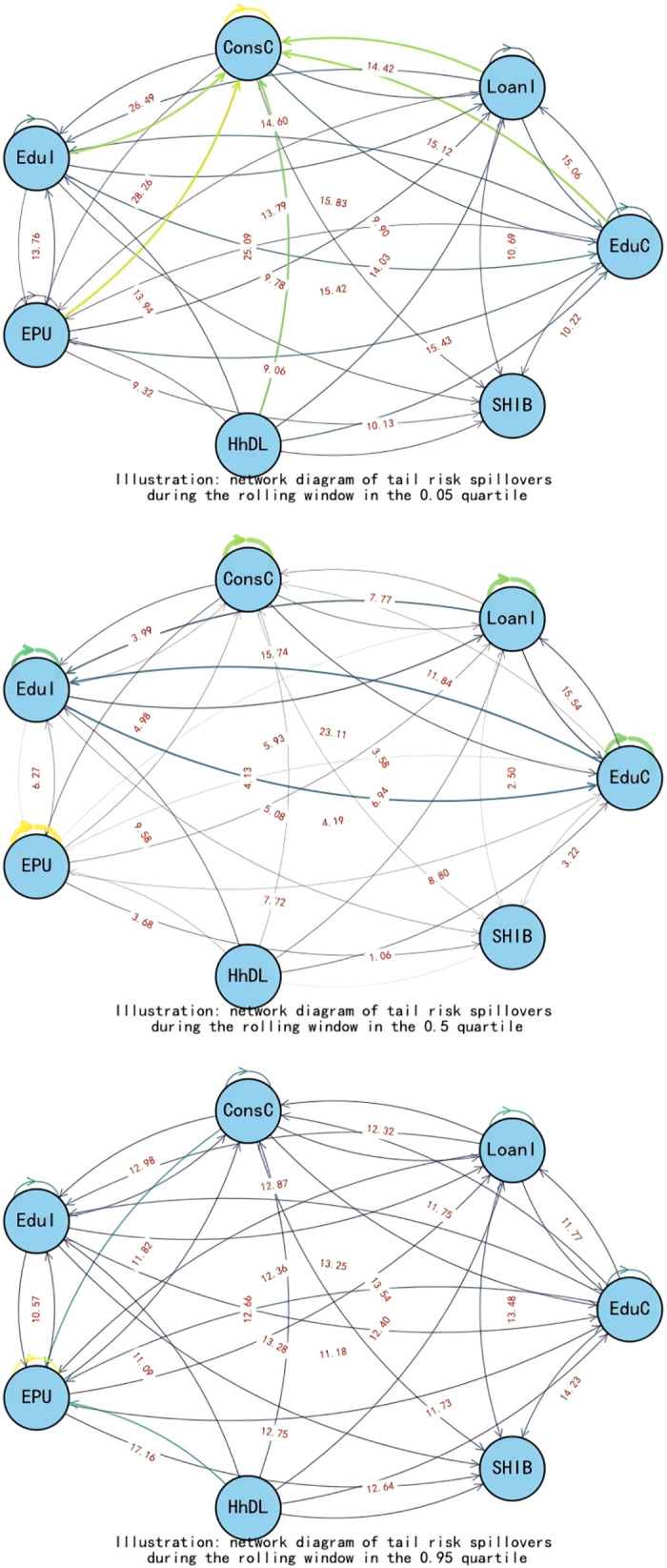
Dynamic spillover effects.

This study uncovers the dynamic interaction characteristics between household education consumption and residents’ credit behavior across different risk levels through a rolling window analysis, as illustrated in Fig 8 and Table 6. Below is a detailed analysis for each risk level:

**Table 6:** Dynamic spillovers.

Tail risk dynamic spillover effect at the 0.05 quantile								
	SHIB	EduC	LoanI	ConsC	EduI	EPU	HhDL	FROM
SHIB	12.74	15.67	13.24	28.12	12.73	8.36	9.12	87.26
EduC	10.22	17.77	13.60	26.31	14.52	7.93	9.64	82.23
LoanI	10.69	15.06	15.69	26.12	15.12	8.10	9.22	84.31
ConsC	9.90	15.12	14.42	30.13	13.32	7.93	9.17	69.87
EduI	9.78	15.83	14.60	26.49	16.70	7.03	9.58	83.30
EPU	9.06	15.42	13.79	28.26	13.76	10.16	9.55	89.84
HhDL	10.13	15.43	14.03	25.09	13.94	9.32	12.06	87.94
TO	59.77	92.54	83.69	160.39	83.40	48.68	56.28	584.74
Inc.Own	72.51	110.32	99.38	190.52	100.10	58.83	68.34	cTCI/TCI
NET	−27.49	10.32	−0.62	90.52	0.10	−41.17	−31.66	97.46/83.53
NPT	2.00	5.00	3.00	6.00	4.00	0.00	1.00	
Tail risk dynamic spillover effect at the 0.5 quantile								
	SHIB	EduC	LoanI	ConsC	EduI	EPU	HhDL	FROM
SHIB	67.18	7.00	5.13	5.12	6.72	5.38	3.46	32.82
EduC	3.22	48.89	13.27	3.71	22.65	2.14	6.13	51.11
LoanI	2.50	15.54	49.43	6.09	19.75	1.39	5.30	50.57
ConsC	3.58	11.84	7.77	51.69	10.47	8.52	6.14	48.31
EduI	5.08	23.11	15.74	3.99	44.50	1.45	6.13	55.50
EPU	7.72	4.19	5.93	4.98	6.27	61.52	9.38	38.48
HhDL	1.06	8.80	6.94	4.13	9.58	3.68	65.82	34.18
TO	23.15	70.49	54.77	28.02	75.44	22.56	36.54	310.98
Inc.Own	90.33	119.38	104.20	79.71	119.94	84.08	102.36	cTCI/TCI
NET	−9.67	19.38	4.20	−20.29	19.94	−15.92	2.36	51.83/44.43
NPT	1.00	6.00	4.00	1.00	5.00	1.00	3.00	
Tail risk dynamic spillover effect at the 0.95 quantile								
	SHIB	EduC	LoanI	ConsC	EduI	EPU	HhDL	FROM
SHIB	16.27	12.32	12.34	12.83	11.42	15.48	19.33	83.73
EduC	14.23	15.79	11.75	12.93	13.60	14.07	17.62	84.21
LoanI	13.48	11.77	17.29	11.81	14.32	12.24	19.09	82.71
ConsC	13.54	11.75	12.32	15.48	11.27	16.26	19.39	84.52
EduI	13.28	13.25	12.87	12.98	16.12	12.95	18.56	83.88
EPU	12.75	11.18	12.36	11.82	10.57	21.32	19.99	78.68
HhDL	12.64	11.73	12.40	12.66	11.09	17.16	22.31	77.69
TO	79.91	72.00	74.05	75.03	72.27	88.16	113.99	575.42
Inc.Own	96.18	87.78	91.35	90.51	88.39	109.48	136.30	cTCI/TCI
NET	−3.82	−12.22	−8.65	−9.49	−11.61	9.48	36.30	95.90/82.20
NPT	4.00	1.00	2.00	2.00	2.00	4.00	6.00	

Low-Risk Level (0.05 quantiles): In a low-risk environment, education expenditure (EduC) and household debt-to-income ratio (HhDL) exhibit significant spillover effects on other variables. Specifically, the spillover effect of education expenditure on consumer confidence (ConsC) reaches 26.31%, indicating that household investment in education can effectively elevate expectations for future economic conditions and enhance market confidence. Concurrently, the influence of the household debt-to-income ratio on the Shanghai Interbank Offered Rate (SHIB) stands at 10.13%, demonstrating the direct impact of changes in household debt on the supply and demand for funds in the credit market. At this stage, the total spillover index (TO) is 584.74, far exceeding the static window value, proving that interactions among variables are more frequent and complex. The net spillover index (NET) reveals that education expenditure and consumer confidence are the primary net spillover transmitters. At the same time, economic uncertainty and credit market interest rates are the leading receivers, highlighting the positive role of education expenditure in stabilizing the economic environment. This analysis serves to validate Hypothesis 2.

Medium-Risk Level (0.5 quantiles): As risk escalates to a medium level, the spillover effects of education income (EduI) and education expenditure on other variables intensify, particularly evident in the 15.74% spillover effect of education income on loan indicators (LoanI). This reflects that increased education income can stimulate credit demand, especially as higher-income households tend to expand their education investments in pursuit of higher returns. Additionally, the spillover effect of education expenditure on education income is 23.11%, suggesting that educational investments may indirectly promote growth in education income by enhancing educational quality. At this stage, the total spillover index decreases to 310.98, indicating a weakened intensity of interactions among variables, yet the significance of education-related indicators becomes more pronounced. The net spillover index reveals that education income and expenditure emerge as the primary net spillover transmitters, further confirming the shifting role of educational activities in the economy.

**Fig 9:**
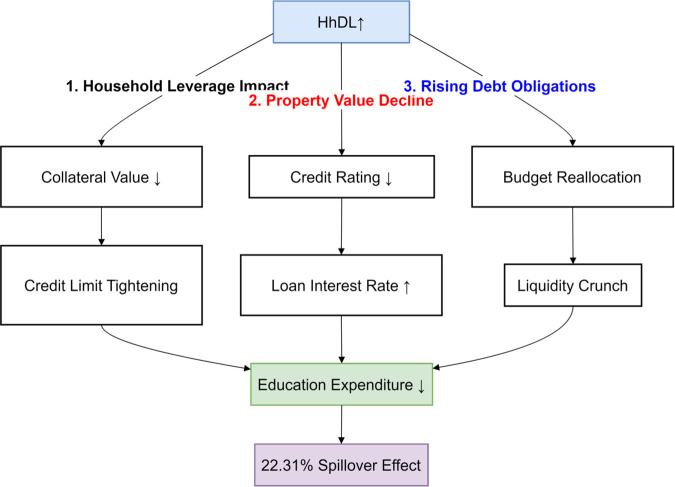
The transmission mechanism of household debt leverage increase on education expenditure and the spillover effect.

High-Risk Level (0.95 quantiles): Under conditions of extreme risk, the spillover effect of the household debt-to-income ratio significantly intensifies to 22.31%, revealing a close linkage between household debt and educational activities. The spillover effects manifest primarily through three interconnected channels (Fig 9). First, as households become more leveraged, financial institutions respond by tightening credit standards, making it harder for families to secure loans—especially for discretionary purposes like education—thereby reducing access to credit. Second, declining property values erode household equity, which not only weakens their borrowing capacity but also prompts a reassessment of spending priorities, often leading to cuts in education expenditure. Finally, rising debt obligations consume a larger share of household income, creating a liquidity crunch that forces families to reallocate resources away from non-essential items such as education toward meeting basic living needs, all of which together amplify the broader economic impact at the micro level.

The spillover effects of the household debt-to-income ratio on education expenditure and education income are 11.73% and 18.56%, respectively, indicating that high debt levels constrain households’ ability to invest in education. The spillover effect of education expenditure on the household debt-to-income ratio is 11.75%, suggesting that increased education expenditure exacerbates the household debt burden. At this stage, the total spillover index approximates the low-risk level at 575.42, demonstrating that even in a high-risk environment, the variables maintain a high frequency of interaction. The net spillover index shows that the household debt-to-income ratio is the primary net spillover transmitter. At the same time, education expenditure and education income are the leading receivers, highlighting the negative impact of household debt on educational decisions. This analysis serves to validate Hypothesis 2.

#### 4.3.3. Analysis of scale effects: The relationship between household education consumption and credit across short-term, medium-term fluctuations, and long-term trends.

**Fig 10:**
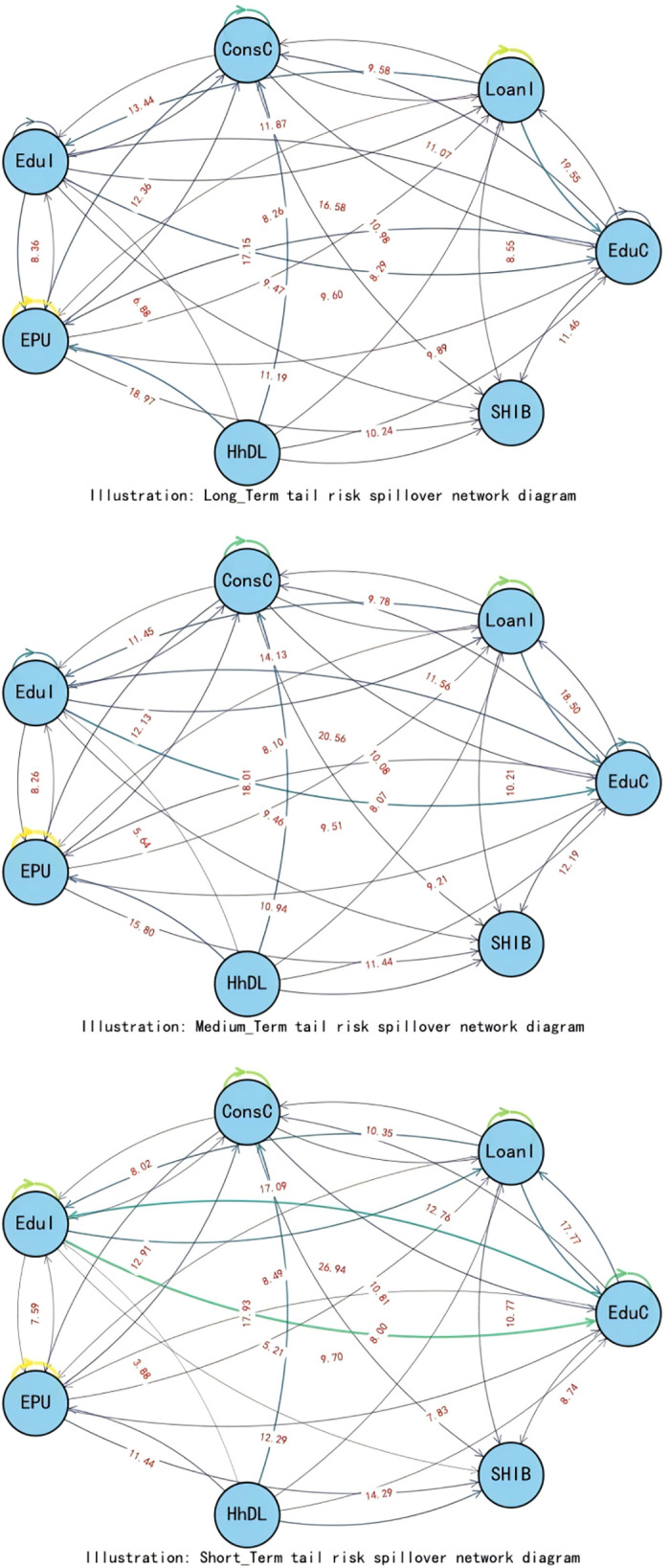
Scale spillover effects.

We uncover the dynamic correlation characteristics between household education consumption and residents’ credit behavior across different time scales (short-term, medium-term, and long-term), as illustrated in Fig 10 and Table 7. Below is a detailed analysis for each time scale:

**Table 7:** Scale spillovers.

Short-term tail risk spillover effect								
	SHIB	EduC	LoanI	ConsC	EduI	EPU	HhDL	FROM
SHIB	34.74	10.88	12.01	11.83	5.54	11.44	13.55	65.26
EduC	8.74	27.90	15.92	11.22	23.02	7.24	5.97	72.10
LoanI	10.77	17.77	31.14	10.15	16.30	7.08	6.80	68.86
ConsC	10.81	12.76	10.35	31.75	7.80	10.97	15.54	68.25
EduI	5.21	26.94	17.09	8.02	32.65	6.64	3.45	67.35
EPU	12.29	9.70	8.49	12.91	7.59	37.35	11.66	62.65
HhDL	14.29	7.83	8.00	17.93	3.88	11.44	36.63	63.37
TO	62.11	85.88	71.86	72.07	64.13	54.80	56.98	467.84
Inc.Own	96.85	113.78	103.00	103.82	96.78	92.16	93.61	cTCI/TCI
NET	−3.15	13.78	3.00	3.82	−3.22	−7.84	−6.39	77.97/66.83
NPT	2.00	6.00	5.00	4.00	3.00	0.00	1.00	
Mid-term tail risk spillover effect								
	SHIB	EduC	LoanI	ConsC	EduI	EPU	HhDL	FROM
SHIB	34.77	10.52	11.08	13.24	5.06	11.96	13.37	65.23
EduC	12.19	19.88	13.51	12.34	16.02	11.07	14.98	80.12
LoanI	10.21	18.50	32.37	9.55	16.44	7.30	5.63	67.63
ConsC	10.08	11.56	9.78	29.39	7.99	12.95	18.24	70.61
EduI	9.46	20.56	14.13	11.45	21.04	10.39	12.97	78.96
EPU	10.94	9.51	8.10	12.13	8.26	39.12	11.95	60.88
HhDL	11.44	9.21	8.07	18.01	5.64	15.80	31.82	68.18
TO	64.32	79.86	64.67	76.73	59.41	69.47	77.14	491.61
Inc.Own	99.09	99.75	97.04	106.12	80.46	108.59	108.96	cTCI/TCI
NET	−0.91	−0.25	−2.96	6.12	−19.54	8.59	8.96	81.93/70.23
NPT	2.00	2.00	4.00	3.00	1.00	5.00	4.00	
Long-term tail-risk spillover effect								
	SHIB	EduC	LoanI	ConsC	EduI	EPU	HhDL	FROM
SHIB	31.39	10.85	12.20	13.11	6.06	12.47	13.92	68.61
EduC	11.46	15.80	11.41	14.07	12.45	15.45	19.37	84.20
LoanI	8.55	19.55	34.48	8.87	17.21	6.61	4.74	65.52
ConsC	10.98	11.07	9.58	25.60	7.97	15.38	19.42	74.40
EduI	9.47	16.58	11.87	13.44	15.74	14.82	18.09	84.26
EPU	11.19	9.60	8.26	12.36	8.36	37.00	13.22	63.00
HhDL	10.24	9.89	8.29	17.15	6.88	18.97	28.58	71.42
TO	61.89	77.53	61.61	78.99	58.93	83.70	88.76	511.41
Inc.Own	93.28	93.33	96.09	104.59	74.67	120.70	117.34	cTCI/TCI
NET	−6.72	−6.67	−3.91	4.59	−25.33	20.70	17.34	85.24/73.06
NPT	2.00	2.00	4.00	3.00	1.00	5.00	4.00	

Short-term (1–4 Periods): In the short term, household education consumption and residents’ credit behavior exhibit significant time-varying characteristics, with frequent spillover effects among variables. The total spillover index of education expenditure (EduC) to other variables reaches 85.88, indicating that changes in EduC substantially impact other economic variables. The spillover effect of the household debt-to-income ratio (HhDL) is also relatively significant, at 63.37, suggesting that fluctuations in household debt levels directly impact credit market interest rates and education expenditure in the short term. The net spillover index reveals that EduC is the primary net spillover transmitter, while economic policy uncertainty (EPU) and HhDL are the leading receivers. This indicates that changes in EduC have a larger impact on other variables in the short term, while EPU and household debt levels are more susceptible to external shocks.

Medium-term (5–8 Periods): Entering the medium term, the interaction between household education consumption and residents’ credit behavior remains significant but somewhat weakened. The total spillover index of education expenditure (EduC) decreases to 79.86, indicating its impact exists but with reduced intensity. The spillover effect of the household debt-to-income ratio (HhDL) is 68.18, maintaining a high level, reflecting the continuing influence of household debt on the credit market and household economic decisions in the medium term. Notably, the spillover effect of education income (EduI) increases to 78.96, suggesting that changes in EduI have a more pronounced impact on other economic variables in the medium term. The net spillover index shows that EduC and EduI become the primary net spillover transmitters. At the same time, consumer confidence (ConsC) and HhDL are the leading receivers, indicating an enhanced influence of education-related factors on economic variables in the medium term.

Long-term (9–12 Periods): In the long term, the interaction between household education consumption and residents’ credit behavior tends to stabilize, but the spillover effects remain significant. The total spillover indices of education expenditure (EduC) and the household debt-to-income ratio (HhDL) are 77.53 and 71.42, respectively, indicating that their long-term impacts on other economic variables remain robust. The spillover effect of education income (EduI) continues to maintain a high level, demonstrating its importance in the long term. The net spillover index reveals that EduC and EduI remain the primary net spillover transmitters. At the same time, consumer confidence (ConsC) and HhDL are the leading receivers, indicating that the influence of education-related factors on other variables is persistent and stable in the long run.

Based on the above analysis, Hypothesis 4 is confirmed.

#### 4.3.4. Comprehensive analysis of total spillover effects.

**Fig 11:**
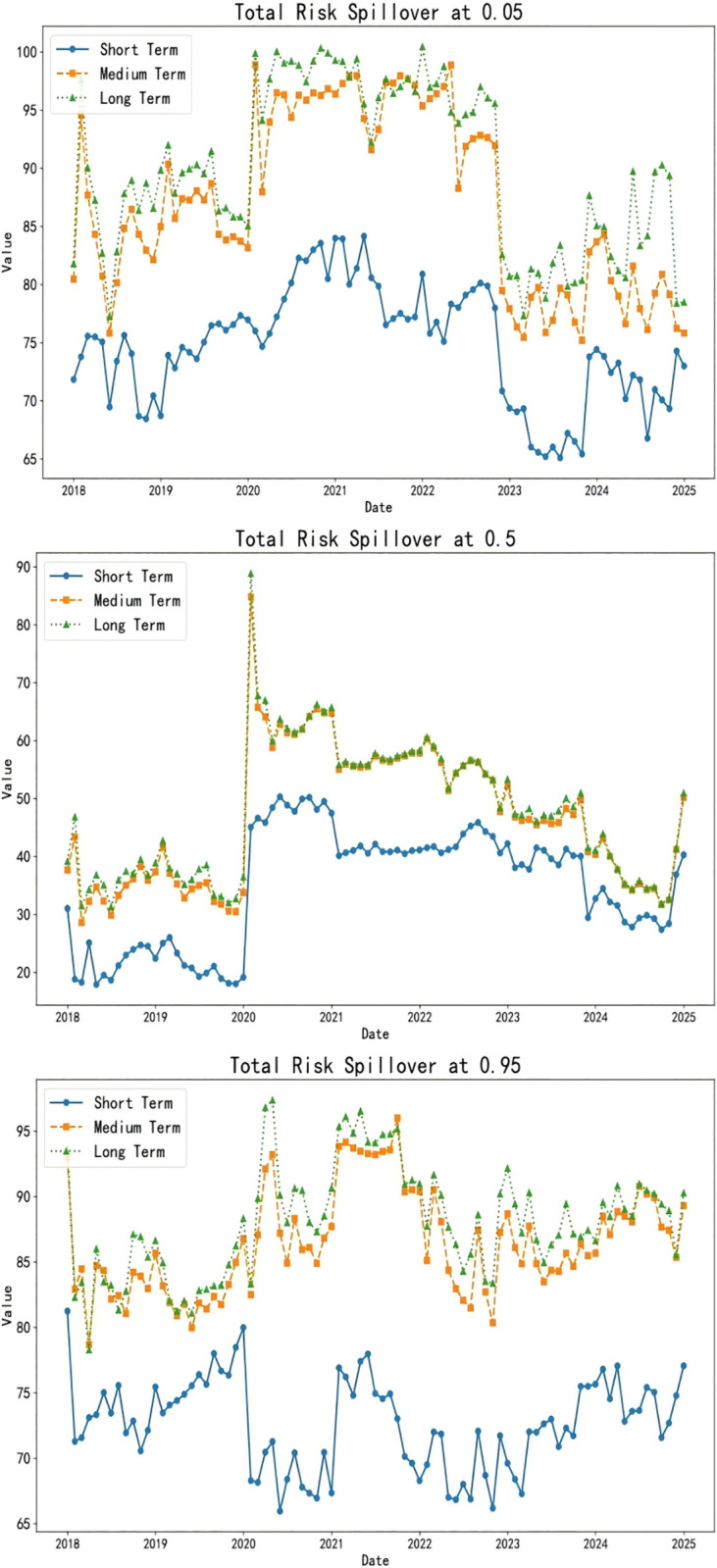
Scale total spillover effects.

To analyze the interaction mechanism between education consumption and credit behavior, a deeper examination of the total tail risk spillover effect is required, as illustrated in Fig 11:

As a crucial component of household resource allocation, the scale of household education consumption is influenced by various factors, including but not limited to household income, educational expectations, and the socio-economic environment. Meanwhile, residents’ credit behavior not only reflects households’ perceptions of their current financial situations but also indicates of future income expectations. According to the life-cycle hypothesis and the permanent income hypothesis, households tend to smooth their consumption paths, potentially relying on credit to meet short-term financial needs, especially in terms of investments in educational resources.

Low-Risk Level (0.05 quantiles): From late 2019 to early 2020, the rapid market response to uncertainty during the initial stages of the COVID-19 pandemic led to a significant increase in short-term volatility. As fiscal stimulus policies were implemented and vaccine development progressed from mid-2020 to early 2021, short-term volatility gradually stabilized; however, long-term volatility remained high, indicating investors’ caution regarding the path of future economic recovery. Since mid-2021, factors such as global supply chain disruptions and energy price fluctuations have continuously elevated long-term risk spillover effects, revealing the global economy’s complexity and uncertainty.

Medium-Risk Level (0.5 quantiles): From late 2019 to early 2020, market tensions significantly increased short-term volatility. As we moved into mid-2020 to early 2021, implementing economic stimulus measures and improvements in pandemic control mitigated short-term volatility, with medium-term volatility remaining relatively stable, demonstrating the effectiveness of policy interventions. However, since mid-2021, long-term volatility has persisted despite the stability in medium-term volatility, particularly when confronted with multiple challenges such as global economic restructuring, climate change, and geopolitical tensions. In these circumstances, long-term risk spillover effects continue to emerge.

High-Risk Level (0.95 quantiles): From late 2019 to early 2020, the market’s heightened sensitivity to significant adverse events resulted in intense short-term volatility. From mid-2020 to early 2021, short-term volatility did not completely dissipate, and medium-term volatility began to rise, revealing structural issues of global economic recovery. Since mid-2021, long-term volatility has peaked, demonstrating vulnerability at high-risk levels due to uneven recovery among global economies, increasing inflationary pressures, and expectations of monetary policy normalization.

Further analysis reveals significant differences in educational investment and credit decision-making among households with different socio-economic backgrounds. For instance, higher-income households are more likely to support their children’s education through borrowing, whereas lower-income households may reduce educational expenditures due to difficulty accessing credit. This analysis serves to validate Hypothesis 3.

**Fig 12:**
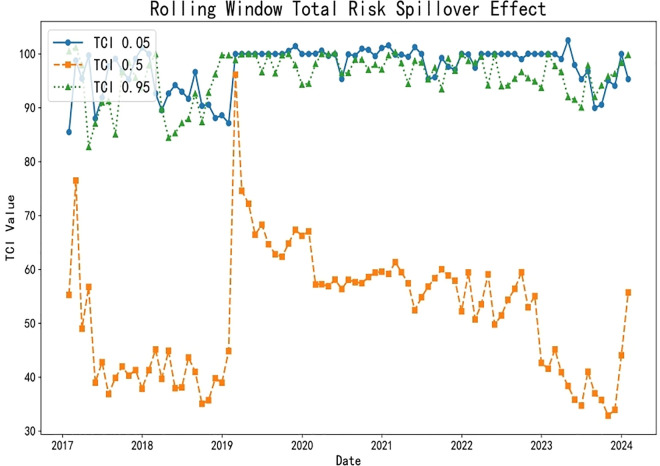
Total spillover effects across different quantiles.

We present Fig 12 to explore the time-varying bidirectional causality between household education consumption and residents’ credit behavior through a dynamic quantile regression model and revealthe underlying heterogeneous mechanisms.

At the low-risk level (0.05 quantiles), we first observe a stable, mutually reinforcing relationship between household education consumption and credit behavior from 2017 to 2018, reflecting family financial planning strategies during stable economic growth. However, with the increase in global economic uncertainty, particularly the intensification of trade tensions, this relationship experienced significant fluctuations from early 2019 to early 2020. The COVID-19 pandemic further exacerbated this trend, resulting in a substantial rise in risks associated with household education investment from mid-2020 to early 2021.

Secondly, at the medium-risk level (0.5 quantiles), the research reveals that the relationship between household education expenditure and access to credit is not static, even during relatively stable economic periods. For instance, from late 2019 to early 2020, due to the deterioration of the external economic environment, households tended to reduce non-essential expenditures, including education investments, thereby affecting credit demand. Conversely, in the early stages of the pandemic, fiscal stimulus measures introduced by governments partially alleviated households’ economic pressures, prompting a rebound in credit market activity.

Lastly, at the high-risk level (0.95 quantiles), our data indicates that household education consumption becomes a pivotal area of focus for family budget adjustments amidst high uncertainty. Particularly from late 2019 to early 2021, as economic challenges intensified, household education expenditure declined markedly, accompanied by increased credit default rates, revealing the profound impact of economic crises on vulnerable groups.

This study utilizes a micro-dataset provided by the National Bureau of Statistics as the sample source. It employs a dynamic quantile regression model to capture the nonlinear relationship between household education consumption and credit behavior as they evolve. The results reveal that household education consumption is influenced by current income levels and closely related to expected future income. Especially during periods of high economic uncertainty, households adjust their credit decisions to cope with potential increases in education expenses.

Fig 12 visually represents how the overall spillover effects evolve over time at various quantiles.

**Fig 13:**
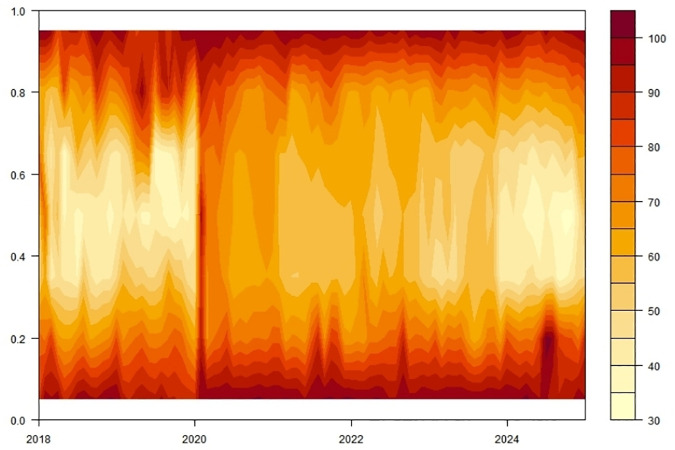
Multi-quantile spillover effects.

As shown in Fig 13, the analysis of spillover effects across different risk levels reveals distinct patterns in household investment behavior in educational capital during the period from 2018 to 2022.

Initially, at lower-risk levels (0.15–0.35 quantiles), the heatmap illustrates that the total spillover effects were relatively low between 2018 and 2019, indicated by lighter colors, suggesting market stability and households’ inclination to maintain steady investments in educational capital. However, from early 2020 to early 2021, amidst the heightened market uncertainty in the initial stages of the COVID-19 pandemic, the total spillover effects notably surged, evident from the darker colors, mirroring households’ behavior in adjusting their credit access and educational capital investment strategies in response to uncertainty.

Furthermore, at medium-risk levels (0.40–0.60 quantiles), the data reveals that the total spillover effects remained relatively stable between 2018 and 2019. However, from early 2020 to early 2021, due to the impact of the pandemic, the total spillover effects similarly increased significantly, indicating a heightened sensitivity of the market towards educational capital investments. Subsequently, from mid-2021–2022, despite a decline, the total spillover effects remained relatively high, reflecting the market’s ongoing concerns about long-term uncertainty.

Lastly, at high-risk levels (0.70–0.95 quantiles), the total spillover effects were relatively low, with minimal fluctuations during 2018–2019. Nevertheless, from early 2020 to early 2021, amidst the shock of the pandemic, the total spillover effects surged dramatically, reflecting households’ substantial adjustments to their credit access and educational capital investment strategies in a high-risk environment. From mid-2021–2022, the total spillover effects remained high, demonstrating households’ heightened sensitivity to long-term economic instability.

#### 4.3.5. In-depth exploration of net spillover effects.

**Fig 14:**
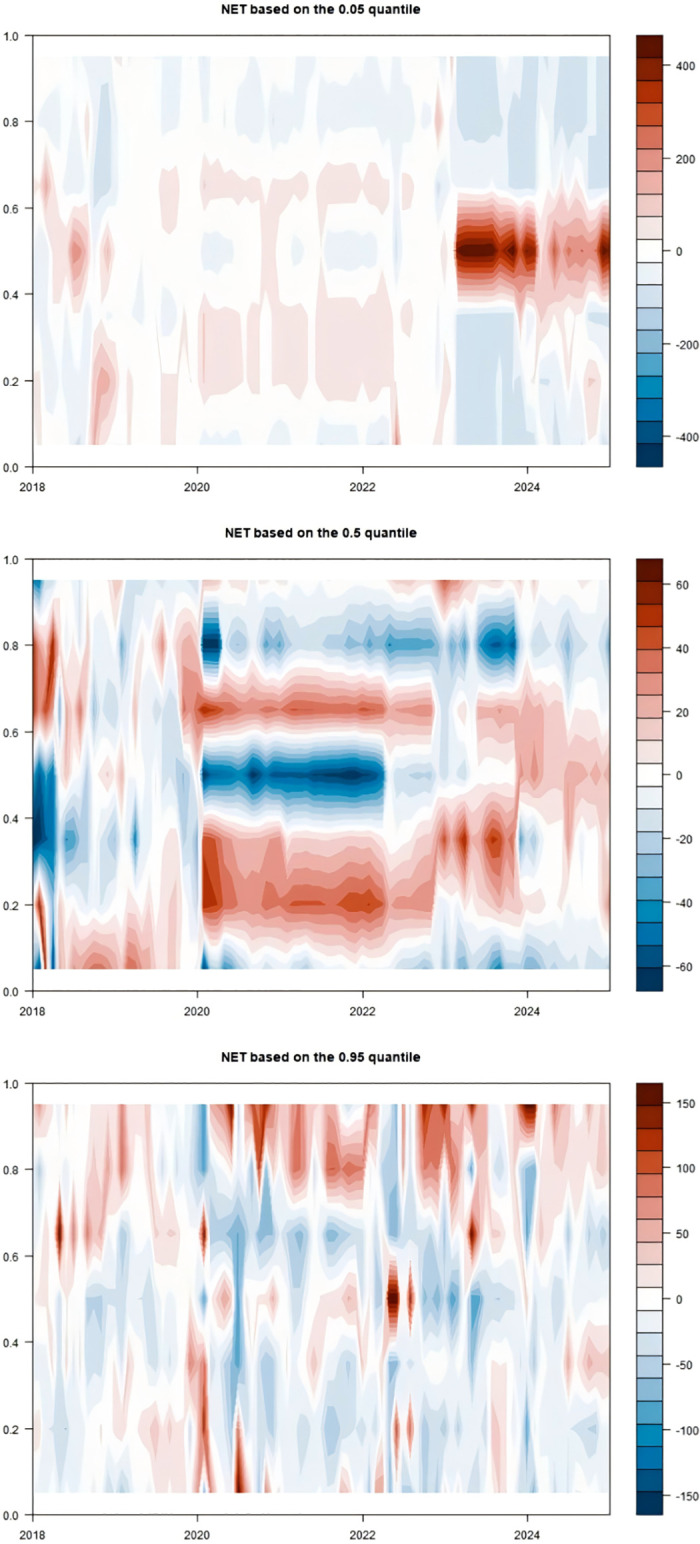
Net spillover effects.

Fig 14 unveils the dynamic changes in the net spillover effects of household educational consumption on the tail risks of household credit behavior across various quantile levels. This analysis is important for understanding the complexity of household financial behavior. In static analysis, household educational consumption is often regarded as a stable influencing factor of household credit behavior. However, results from dynamic analysis indicate that when the market experiences positive shocks of a certain magnitude, particularly under conditions of low-risk levels (0.05 quantiles), medium-risk levels (0.5 quantiles), and high-risk levels (0.95 quantiles), the tail risk spillover effects of household educational consumption on household credit behavior significantly intensify. Notably, this effect was particularly pronounced during 2019 and 2020, which may be related to the increased global economic uncertainty, escalating trade tensions, and the outbreak of the COVID-19 pandemic. These findings suggest that examining only the tail risk spillover effects of household educational consumption in extreme and normal states may lead regulators to overlook its importance in household financial decision-making, thereby relaxing supervision over credit behaviors related to household educational consumption and posing potential financial risks.

### 4.4. Robustness tests

We analyzed system stability using a VAR model in the robustness tests, verifying the reliability and validity of our conclusions by checking if the model’s characteristic roots lay within the unit circle. Then, we performed dynamic analyses with a TVP-VAR-DY model to capture the time-varying parameters and check the robustness of our results. Combining these two models, we accounted for dynamic data features and potential structural changes, ensuring the stability and universality of our findings across different economic settings. The consistent validation of the impact relationship and heterogeneity mechanism between household education expenditure and residents’ credit behavior in both the VAR stability tests and the TVP-VAR-DY dynamic analyses demonstrates the robustness and reliability of our conclusions.

## 5. Conclusions and policy implications

This study establishes a novel analytical framework integrating TVP-SV-VAR and QVAR-DY methodologies to investigate the dynamic interdependencies between household education expenditure and resident credit behavior. Our longitudinal analysis of urban Chinese households(2015–2024) provides robust empirical evidence of time-varying bidirectional causality between these variables, characterized by significant quantile-specific heterogeneity and asymmetric spillover effects across economic cycles.

### 5.1. Four pivotal findings emerge

Dynamic Bidirectional Causality and Household Adaptation. We identify countercyclical fluctuations in the bidirectional causal intensity. Following positive shocks to education expenditure, households initially increase borrowing to meet short-term funding needs. However, this credit dependency diminishes over time, demonstrating household financial adaptability. Conversely, enhanced credit accessibility stimulates educational investment, particularly where resources are scarce, or expected returns are high, though this effect stabilizes as debt accumulates.

Risk-Tiered Heterogeneity. The relationship exhibits distinct quantile-based patterns. Low-risk households exhibit education expenditure-driven credit dependency. In contrast, high-leverage households face constrained educational investment capacity despite heightened sensitivity to expenditure changes, reflecting the impact of accumulated debt. The spillover effects of education expenditure and household leverage ratios on other variables are particularly pronounced across these risk tiers.

Heterogeneous Moderating Mechanisms. Key factors moderate the strength of the bidirectional link. Higher disposable education income amplifies the effect of education expenditure on credit demand. Conversely, rising credit interest rates and heightened economic policy uncertainty suppress these linkages. Households respond to uncertainty by initially reducing education expenditures to mitigate financial pressures. However, this effect attenuates over time and is consistent with intertemporal resource allocation under the life-cycle hypothesis and liquidity constraints theory.

Temporal Spillover Asymmetry. Significant asymmetries exist across time horizons. Education expenditure exerts more substantial short-term impacts on credit markets than the long-term feedback effects on expenditure itself, with policy transmission lags approximating one year. While interaction patterns stabilize in the long run, significant linkages persist.

These findings advance household finance theory by elucidating how credit accessibility and human capital investment interact through nonlinear temporal pathways and are differentially moderated by household characteristics and macroeconomic conditions. They offer critical insights for designing tailored regulatory interventions at distinct risk tiers: enhancing education credit facilities during stability, strengthening financial literacy and debt management amid moderate uncertainty, and implementing macroprudential safeguards against debt-driven vulnerabilities during crises. Future research should incorporate cross-cultural comparisons and machine-learning approaches to generalize these dynamics.

#### 5.1.1. Practical impacts.

Educational expenditure, a key part of household budgets, affects children’s educational quality and family financial health. Research shows that when families anticipate higher education costs, they plan finances, possibly via increased savings, consumption structure adjustments, or credit support. For instance, some families may cut non-essential entertainment spending to fund children’s education. Families become more cautious about education expenditure during periods of high economic uncertainty, like global financial crises or pandemic shocks. Many weigh the long-term returns of education against short-term financial pressures, with some delaying or reducing education spending to ensure basic living needs and financial stability.

The growth in educational consumption boosts credit demand, particularly for education loans and credit card installment products. Financial institutions respond by offering more education-targeted credit products and enhancing risk management with stricter loan approval standards. Household credit behavior, in turn, affects credit market supply-demand dynamics and interest rates. A surge in education loan applications can raise loan rates, influencing borrowing costs and education investment decisions.

Economic policy uncertainty also impacts household education consumption and credit behavior. For example, during tax policy adjustments, families may revise education expenditure plans based on new tax incentives to reduce educational costs. Government education subsidy and credit support policies play crucial roles. Policies like interest subsidies for education loans and special educational funds can alleviate families’ educational burdens, boost their enthusiasm for education investment, and stimulate credit market activity.

#### 5.1.2. Theoretical implications.

In family economics, this study offers a new perspective by examining the relationship between educational consumption and credit behavior dynamically and heterogeneously. Traditional theories mainly focus on family consumption and saving behaviors. This study incorporates credit behavior, revealing how families balance consumption, savings, and credit in education investment. It enriches the theoretical understanding of family economic decisions. Moreover, the study shows that educational consumption decisions are not only based on current income and expenditure but also influenced by future income expectations and economic policy uncertainty. This provides theoretical support for refining family economic decision-making models.

In behavioral finance, this study explores families’ risk preferences and behavioral traits in educational consumption and credit decisions, aligning with behavioral finance theories. For instance, families tend to be risk-averse under economic uncertainty, reducing education investment and credit scale to lower financial risks. The study also highlights the impact of irrational family behaviors, such as overconfidence and herd behavior, on credit markets and the macroeconomy. This offers empirical support for applying behavioral finance theories in family finance.

In the economics of education, this study explores the interaction between educational consumption and family financial conditions, opening new research avenues. It shows that educational consumption is a human capital investment closely related to family financial health. This is important for analyzing educational investment returns and equity and provides comprehensive theoretical support for education policy-making. The study also emphasizes the interconnection between education and financial systems by revealing the relationship between educational consumption and credit behavior. It points out that changes in education and financial policies can influence each other, affecting families’ education investment ability and financial market operations.

### 5.2. Policy recommendations

Based on this study’s findings, the following refined policy recommendations are proposed to optimize the interaction between household education expenditure and resident credit behavior, thereby fostering economic stability and household financial health across varying risk environments:

Under low-risk conditions. Policymakers should prioritize sustained investment in education. This entails increasing the absolute level of public funding allocated to education and explicitly raising its proportion within the total government budget. Resource allocation mechanisms must be strategically optimized to ensure equitable access, directing incremental funds and targeted programs towards rural and economically disadvantaged regions to narrow urban-rural and regional educational disparities systematically. Concurrently, financial regulators should actively encourage and facilitate financial institutions in developing tailored educational finance solutions. This includes designing education-specific loan products with flexible repayment schedules (e.g., grace periods aligned with graduation, income-based repayments) and preferential interest rates alongside dedicated education savings plans offering competitive returns or government-matching contributions. Crucially, efforts must be made to lower participation barriers, such as simplifying application processes and relaxing collateral requirements for qualifying low-to-middle-income families seeking education loans.

Under medium-risk conditions. Policy focus should shift towards enhancing efficiency, quality, and household resilience. Governments must strengthen oversight of educational quality and resource utilization efficiency. This involves establishing a robust, multi-dimensional education quality evaluation system incorporating standardized learning outcomes, stakeholder feedback, and resource efficiency metrics. Regular, transparent assessments of educational institutions should be mandated to foster accountability and drive continuous improvement. Simultaneously, comprehensive initiatives to bolster household financial capacity are essential. Nationwide financial literacy campaigns should be rolled out through diverse channels (community workshops, school curricula integration, digital platforms) to improve understanding of credit products, budgeting, risk management, and long-term financial planning related to education financing. These programs should empower households to make informed decisions about education spending and credit usage, mitigating impulsive borrowing and promoting sustainable financial behavior.

Under high-risk conditions. Policymakers must adopt a dual approach focused on systemic risk mitigation and household support. Establishing a dedicated household debt risk monitoring and early warning system is paramount. This system should track key indicators like debt-to-income ratios, delinquency rates specifically for education loans, and credit accessibility metrics, enabling timely identification of emerging vulnerabilities and triggering pre-emptive policy responses. Financial regulators must intensify supervision of lending practices within the education credit market, enforcing strict rules against predatory lending, usurious interest rates, and irresponsible over-extension of credit, particularly for vulnerable households. Concurrently, swift and decisive macroeconomic stabilization policies are critical. Direct income support (e.g., targeted cash transfers), large-scale employment protection and creation programs, and temporary tax relief can significantly improve household income stability and employment security. This enhanced financial buffer enables families to maintain essential education expenditures and meet existing debt obligations during heightened economic stress.

### 5.3. Research limitations

This study acknowledges several constraints in scope and external factor integration: The empirical analysis primarily reflects urban households in developed coastal regions, with insufficient representation of rural and western communities, potentially limiting generalizability to geographically diverse populations. Key policy shifts were not fully incorporated—specifically the 2018 amendments to the Private Education Promotion Law influencing private education investment, the heterogeneous local implementations of the 2021 “Double Reduction” policy, and the 2022 Commercial Bank Internet Lending Regulations tightening credit accessibility—alongside significant market disruptions including the 2017–2019 P2P lending collapse affecting alternative education financing and the 2023 local government bond defaults impacting public education funding. These omissions may affect the comprehensiveness of the credit-education causality interpretation under structural economic transitions.

### 5.4. Future research directions

In light of the limitations of this study, future research can be expanded and deepened in the following aspects.

First, data sources’ time span and scope should be broadened to enhance data diversity and representation. This boosts the universality of research conclusions and better captures the changing characteristics of family educational consumption and credit behavior across different economic cycles and policy environments.

Second, incorporate more external factors into the analysis, such as policy changes, macroeconomic conditions, and sociocultural elements. These factors may significantly impact family educational consumption and credit behavior. Delving into their mechanisms can offer a more comprehensive understanding of the relationship between the two. For example, research could focus on how government educational subsidy policies and credit policies influence family decisions and how macroeconomic fluctuations alter family educational investment and credit behavior.

Third, more sophisticated models and methods, such as nonlinear models and machine-learning algorithms, should be employed to improve the accuracy and comprehensiveness of the analysis. Nonlinear models can better capture complex data relationships, while machine-learning algorithms can handle many variables and complex data structures, uncovering potential patterns and correlations.

Finally, further exploration of the impact of different family structures, income levels, and regional differences on the relationship between educational consumption and credit behavior is needed to provide more targeted policy recommendations. For instance, studying the differences in educational consumption and credit behavior between low-income and high-income families and regional disparities caused by factors like economic development levels and the distribution of educational resources is highly significant for devising precise educational and credit policies. Meanwhile, future research can emphasize policy analysis by exploring ways to optimize the relationship between family educational consumption and credit behavior through policy intervention, thereby promoting educational equity and family financial well-being.

## Supporting information

S1 Appendix Time - varying Granger causality test. (DOCX)
