# Supplementary material for: The time-varying bidirectional causal relationship between household education expenditure and resident credit behavior: Dynamic quantile evidence and heterogeneous mechanisms
Source: PLoS One. 2025 Aug 13;20(8):e0329213. doi: 10.1371/journal.pone.0329213 (PMC12349124; doi:10.1371/journal.pone.0329213)
Supplement: S1 Appendix — (DOCX) [file pone.0329213.s001.docx]

**S1 Appendix**

The rest of the time-varying Granger causality test plots in the text.


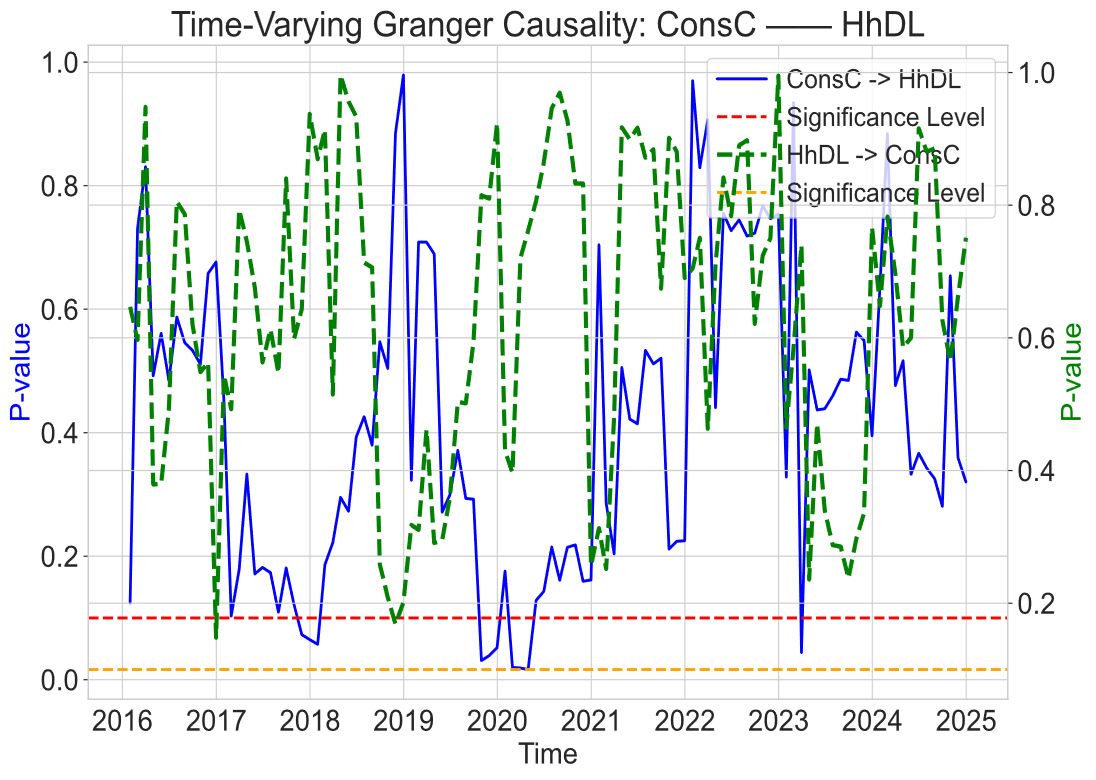

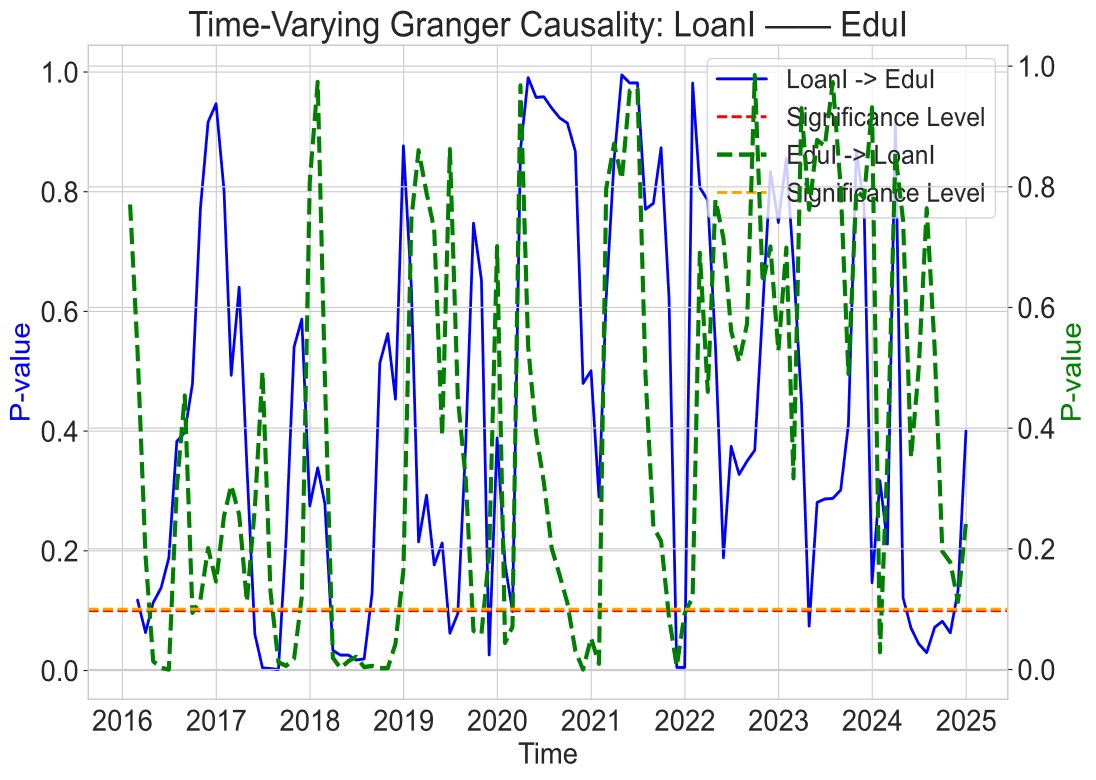

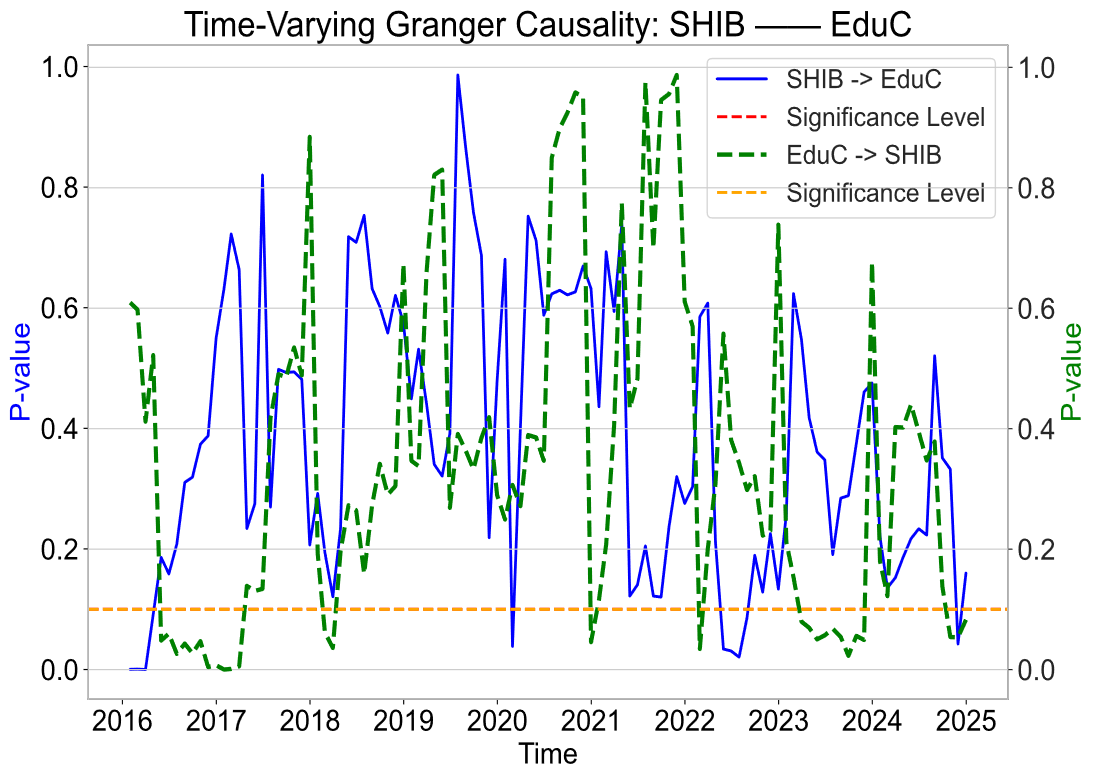

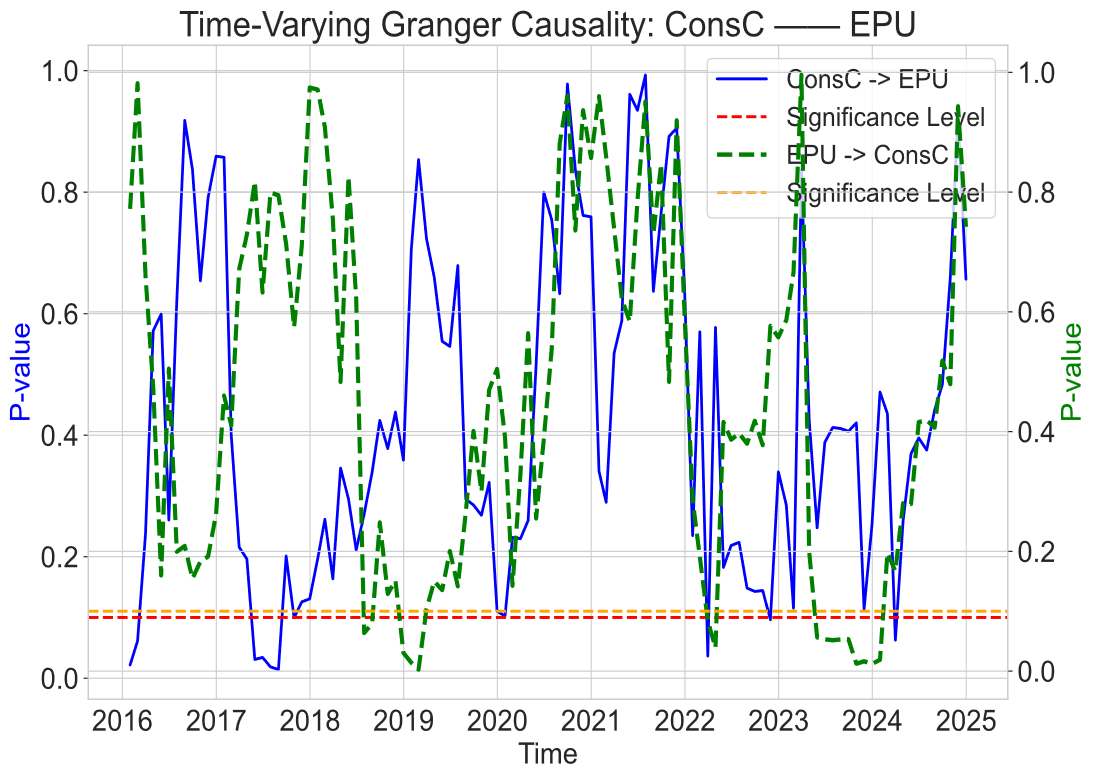

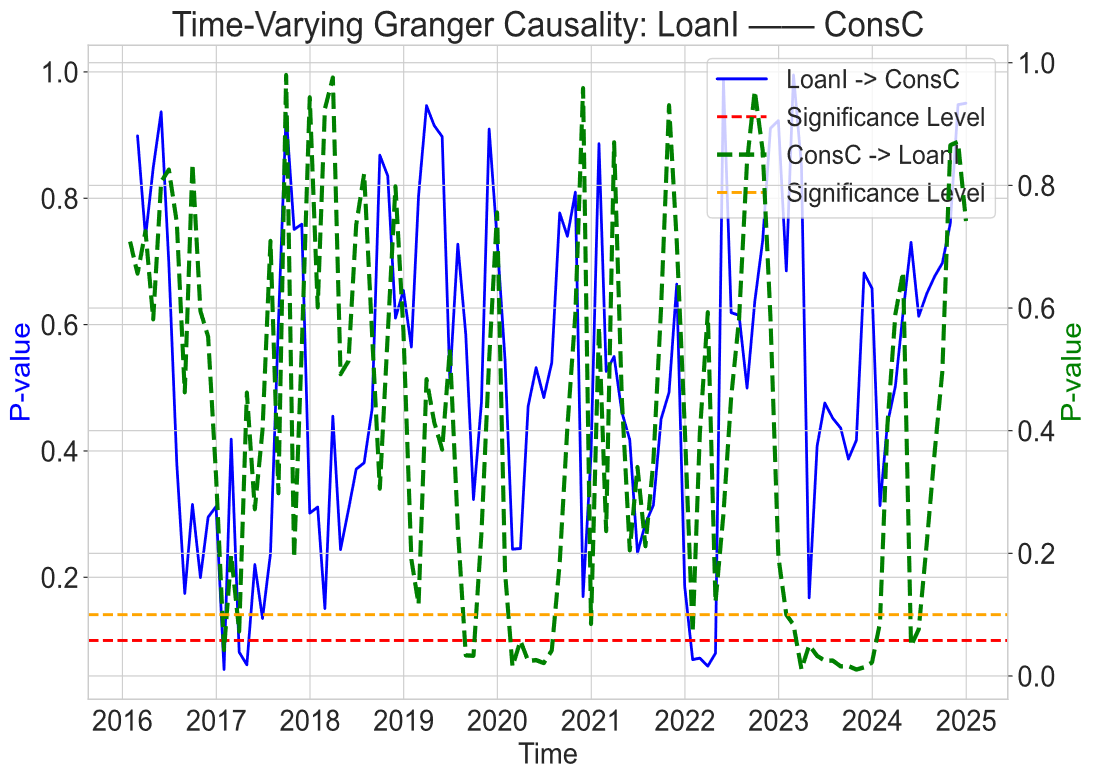

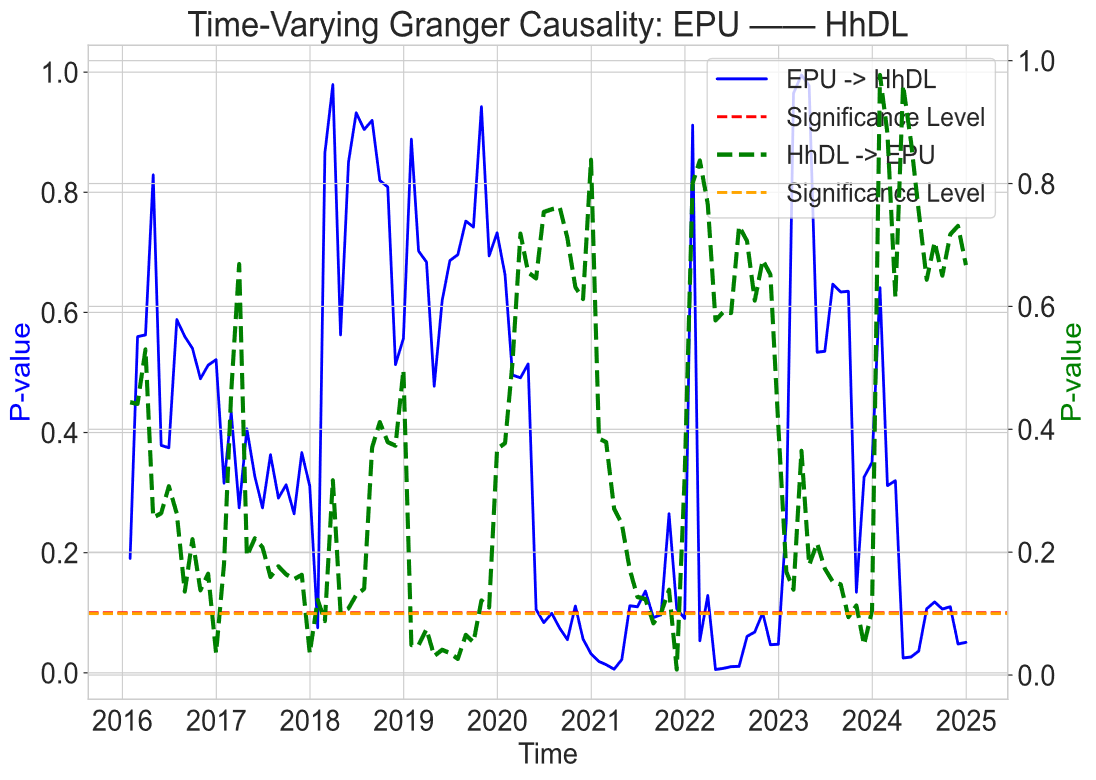

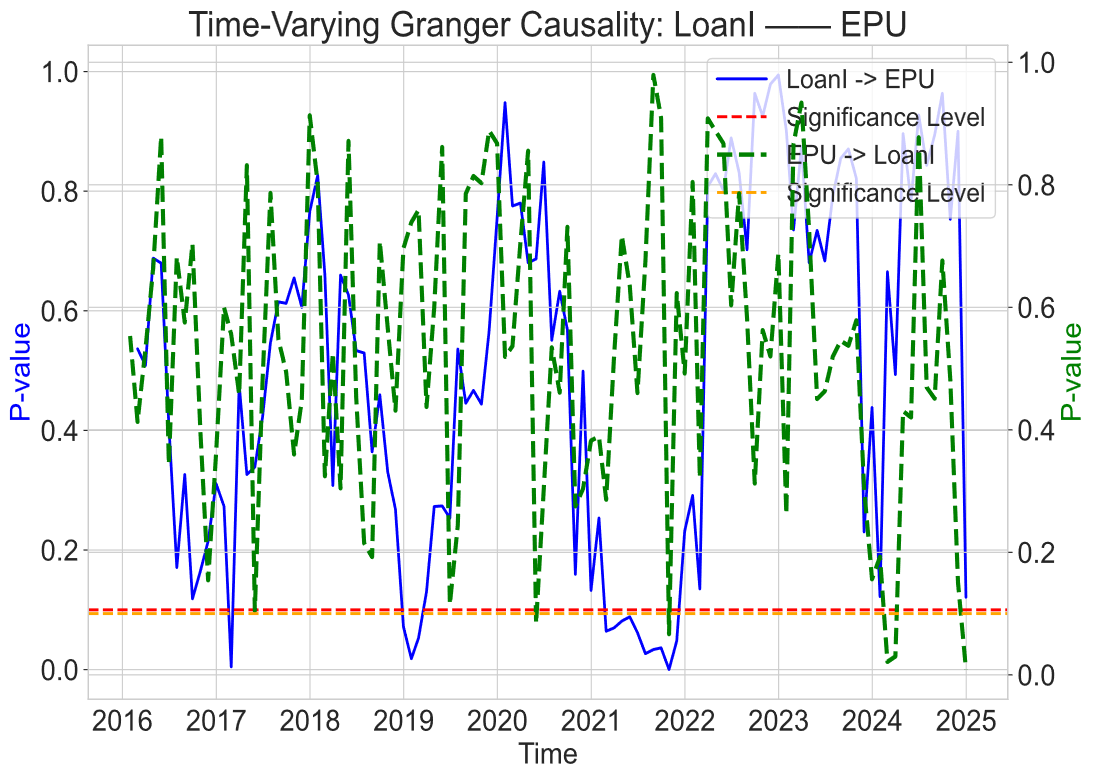

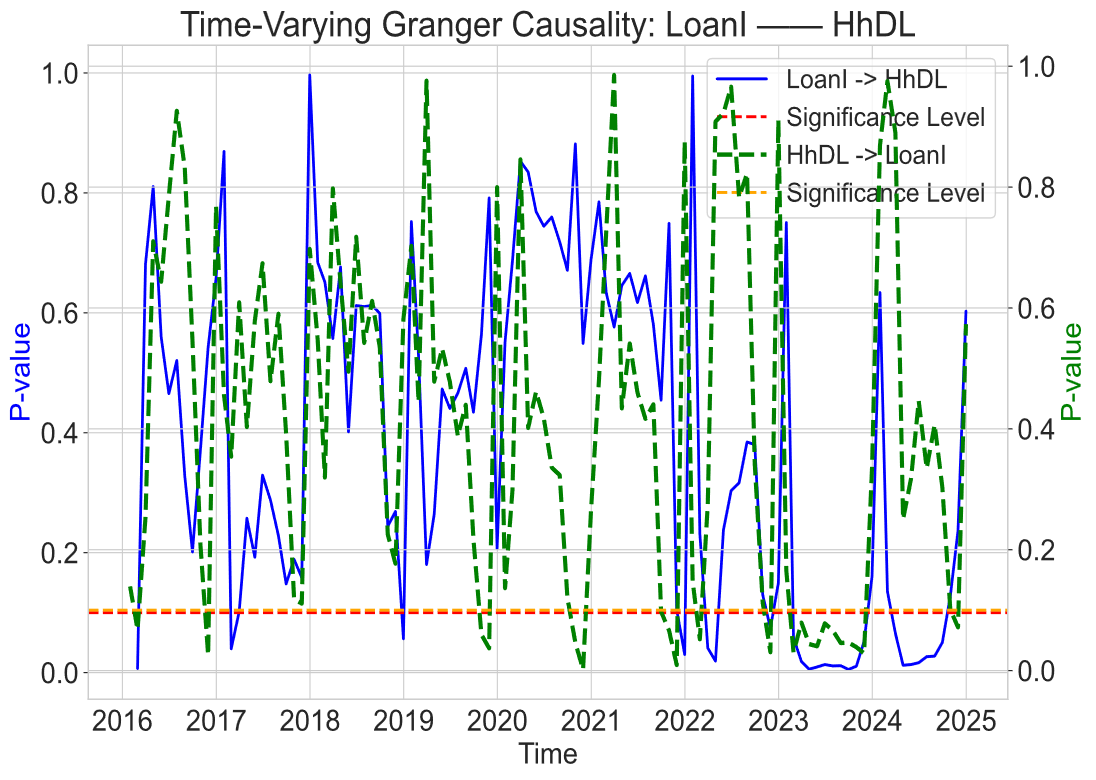

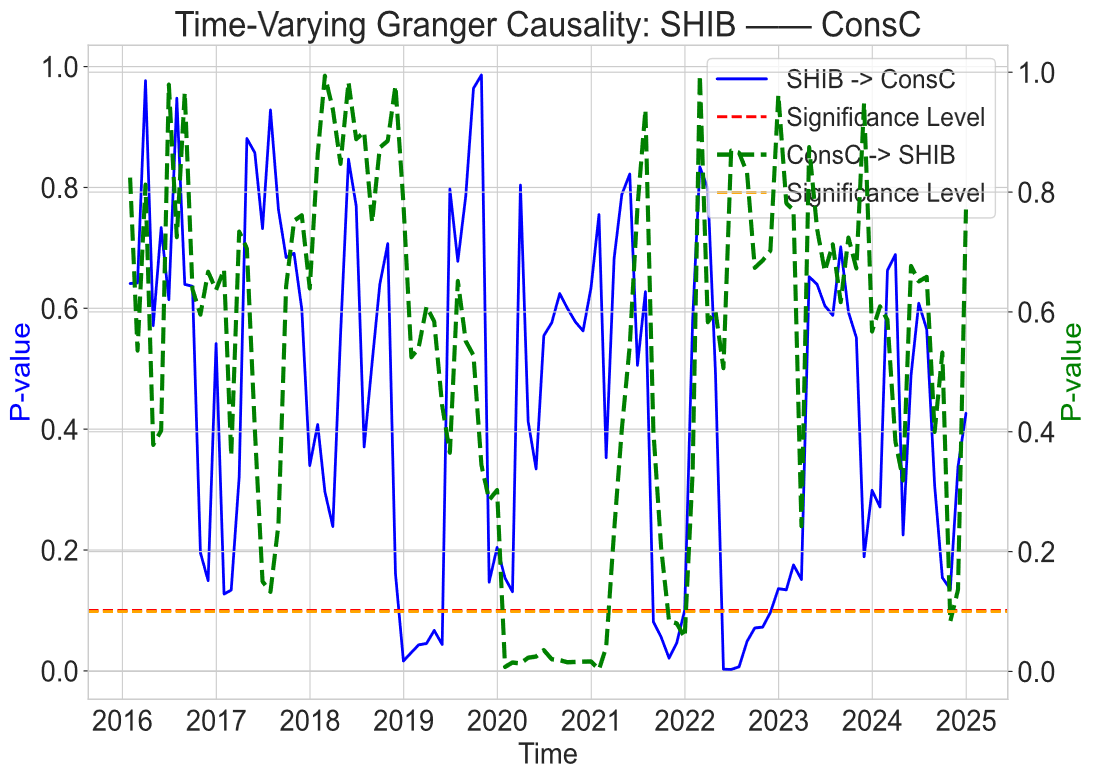

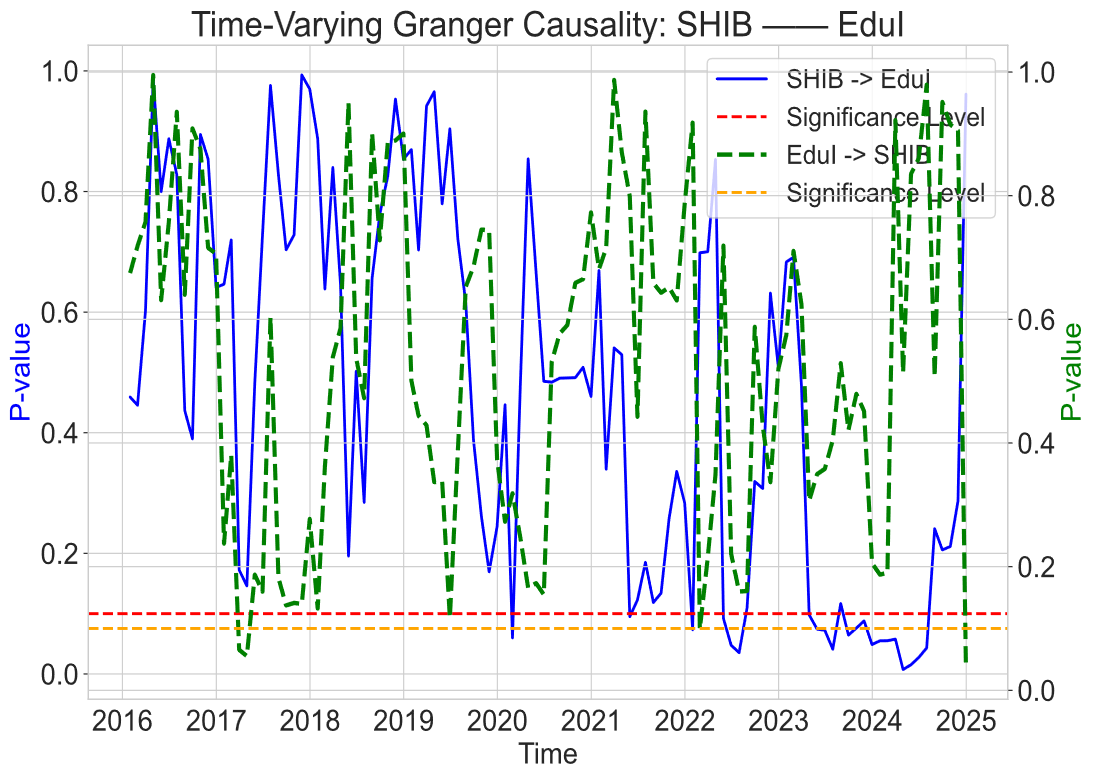

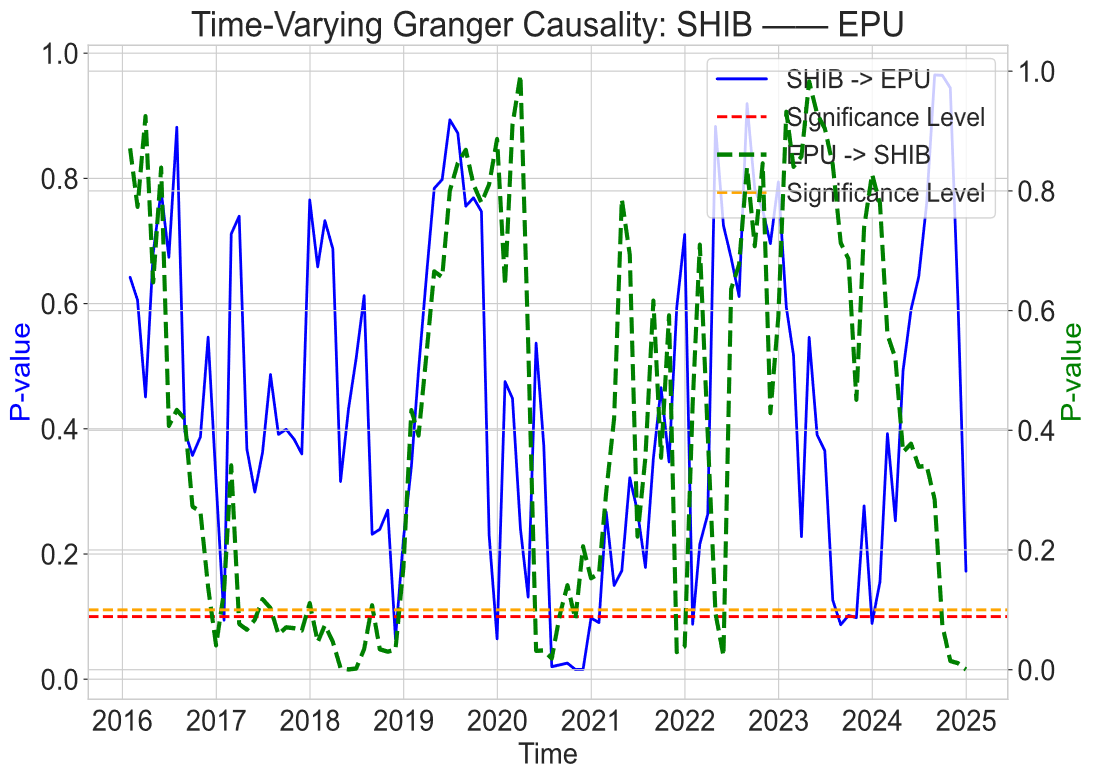

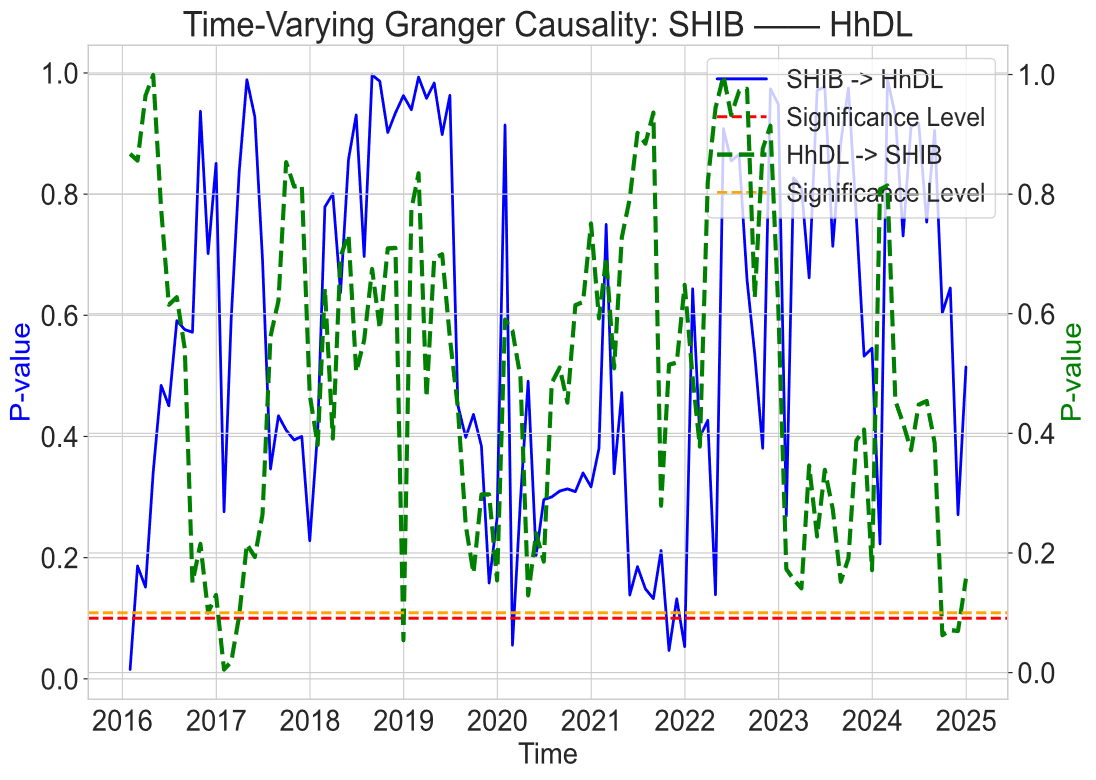

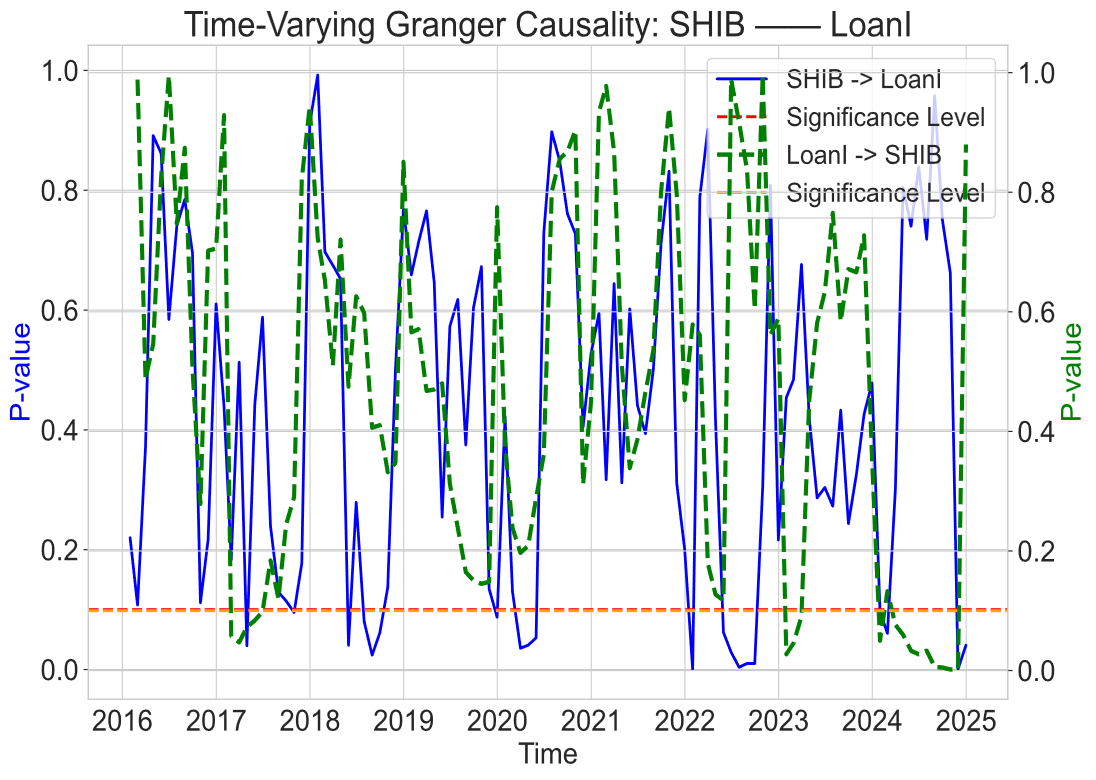


Appendix Fig 1: Time-varying Granger causality test

Appendix Fig A1: Full Set of Time-Varying Granger Causality Test Results (Complementary to Fig 2).
